# General Patterns of Biocompatible Microemulsions Formed with Di-rhamnolipid and Alkanediols

**DOI:** 10.1021/acs.langmuir.6c03072

**Published:** 2026-07-25

**Authors:** Florian Trummer, Rika Raff, Sylvain Prévost, Thomas Sottmann

**Affiliations:** † Institute of Chemical Chemistry, 9149 University of Stuttgart, Pfaffenwaldring 55, 70569 Stuttgart, Germany; ‡ Institut Laue-Langevin − The European Neutron Source, 71 Avenue des Martyrs, F-38042 Grenoble, France

## Abstract

Sustainable biosurfactants, such as rhamnolipids (RL), have been attracting increasing attention because of their enhanced biocompatibility and high biodegradability compared to conventional synthetic surfactants. To enable large-scale application of rhamnolipids, such as cleaning or washing, we studied symmetric, biocompatible microemulsions of the type H_2_O (KCl brine)–isopropyl myristate (IPM)–di-RL (Rha_2_C_10_C_10_)–alkane-1,2-diol. We present a systematic study on the influence of major formulation parameters, namely temperature, salinity, pH, and co-surfactant chain length, on the phase behavior and nanostructure. At pH = 8.0 ≫ p*K*_a_, Rha_2_C_10_C_10_ behaves similarly to double-tail anionic surfactants, including AOT, and does not form oil-continuous microemulsions at low salinities. At higher salinities, a classical fish phase diagram with a three-phase body was observed, implying that the addition of octane-1,2-diol causes an inversion of the amphiphilic film curvature from being curved around IPM to around brine. Lowering the pH to pH = 6.0 ∼ p*K*_a_ leads to the partial protonation of the di-RL and a more hydrophobic behavior. Longer chain alkanediols substantially improve the solubilization efficiency of the amphiphile mixture. Small-angle neutron scattering (SANS) measurements showed an interaction peak at low *q* with values of the amphiphilicity factor *f*_a_ around −0.7, confirming the presence of well-structured microemulsions. Data analysis at high *q* with a modified Porod law revealed surprisingly low values of blurriness *t*, in view of the di-RL’s large headgroup, and a geometrical factor *a* significantly larger than predicted by models of the bicontinuous structure. Both observations might be linked to a scattering contribution of the bulky sugar head groups. Finally, at high pH and low salinity, an additional high-*q* scattering feature is observed, which could be related to the packing of the rhamnose moieties or to the local segregation of di-RLs and alkane-1,2-diols in the amphiphilic film.

## Graphical abstract

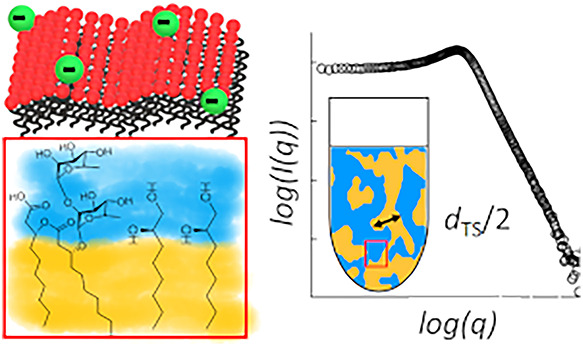

## Introduction

In recent years, biosurfactants have been increasingly under the spotlight of both industrial and academic research groups for their potential to partially replace conventional, petrochemical surfactants. Among other attributes, biosurfactants have been shown to possess enhanced biodegradability^1^ and biocompatibility,^2^ making them interesting for applications in personal care products.^3^ However, the properties and characteristics of this diverse class of amphiphiles are an active topic of research and far from being understood.^4^ Rhamnolipids, a class of glycolipids produced by, e.g., the bacterium *Pseudomonas aeruginosa*, are among the most commonly studied biosurfactants and have become increasingly available on an industrial scale in recent years.^5,6^ Depending on the number of rhamnose units in the headgroup, a distinction is made between mono- (Rha_1_C_10_C_10_) and di-rhamnolipids (Rha_2_C_10_C_10_). As a consequence, a substantial amount of research has focused on the aqueous self-assembly of rhamnolipids^7–9^ as well as their mixing behavior with other surfactants in aqueous solution,^10–12^ revealing intriguing properties that depend on many formulation parameters, including surfactant concentration, temperature, salinity, or pH. The influence of the last two parameters can be traced back to the rhamnolipids’ carboxylic group with a p*K*_a_ of 5.6,^13^ leading to an increasingly deprotonated, charged headgroup at high-pH conditions that greatly influences the surfactant’s interfacial properties and self-assembly. As a consequence, the di-rhamnolipid Rha_2_C_10_C_10_ studied in this work forms a lamellar phase in an aqueous environment at high concentrations at pH ≈ p*K*_a_, whereas at pH ≫ p*K*_a_, a hexagonal phase is observed.^8^ Other studies on both mono- and di-rhamnolipids have demonstrated its pH-controllable foaming properties.^14^ Rhamnolipids may therefore be viewed as an interesting, synergistic edge case between nonionic alkyl polyglucoside sugar surfactants on the one hand and ionic double-tail surfactants, including AOT, on the other hand. Depending on the chosen formulation or desired application, either the nonionic or the ionic characteristics of rhamnolipids can be accentuated, while still preserving the aforementioned beneficial properties.

It is this versatility in combination with excellent enzyme compatibility^15,16^ that makes rhamnolipids very interesting for cosmetic and washing applications, as well as multicomponent formulations based on microemulsions. In these complex fluids, two immiscible liquids, usually water and a nonpolar oil, form a thermodynamically stable, one-phase mixture when a suitable amphiphile is added. When applied, rhamnolipids, being a true surfactant, will reduce the interfacial tension between water and oil, leading to the formation of nanostructured water/oil domains that are separated by a well-defined amphiphilic film, which distinguishes state-of-the-art microemulsions^17,18^ from so-called surfactant-free ones.^19^ In the former, depending on the curvature of the interfacial film, either oil-in-water (O/W), water-in-oil (W/O), or bicontinuous structures composed of interwoven water and oil domains can be formed. The largely hydrophilic nature of rhamnolipids usually necessitates the use of hydrophobic co-surfactants to tune the curvature of the amphiphilic film and enable the formation of bicontinuous or water-in-oil structures, a property that is also known from microemulsions formed with the closely related alkyl polyglucoside surfactants.^20–22^ Recent review articles summarize the results obtained in previous studies on rhamnolipid-stabilized microemulsions.^23,24^ Notable examples include a study by Nguyen, Sabatini, and co-workers, in which water–toluene-based microemulsions stabilized by a mono- and di-rhamnolipid mixture were found to undergo phase inversion upon increasing the salinity, whereas formulations with more hydrophobic oils did not invert.^25^ The same authors also investigated the characteristic curvature^26^ of short-chain (C_8_C_8_) mono/di-RL mixtures,^27^ further showcasing the ionic, hydrophilic nature of rhamnolipids. Xie and co-workers investigated microemulsions formed in brine/alkane/rhamnolipid/alkanol systems^28,29^ and showed the influence of the monohydroxy alcohol chain length on the phase behavior and the formation of liquid crystalline mesophases. More recent contributions have investigated more complex and application-focused systems such as rhamnolipid-containing glycerol/diesel microemulsions,^30^ where a substantially increased glycerol solubilization capacity compared to traditional nonionic surfactants was found. The biocompatibility of rhamnolipids was explored in a study on fruit-preserving microemulsions, which suggested a synergistic antimicrobial effect of essential oils and rhamnolipids.^31^ A further notable study showed the potential of rhamnolipid-based W/O microemulsions for the enzymatic hydrolysis of waste frying oils and demonstrated a significant increase in both the hydrolysis degree and hydrolysis rate when compared with formulations based on traditional surfactants. The authors concluded that this beneficial behavior is directly linked to the high enzyme compatibility of rhamnolipids.^32^

The aim of this study is to extend previous studies on rhamnolipid-stabilized microemulsions by systematically investigating the influence of a wide range of parameters on the phase behavior and nanostructure of symmetric formulations, i.e., those containing equal amounts of brine and oil. While asymmetric, i.e., oil-rich or water-rich formulations are of interest for practical applications, it has been shown that knowledge of the phase behavior of symmetric microemulsions allows for the prediction of the properties of asymmetric formulations, e.g., phase behavior, interfacial tension, and microstructural length scale.^22,33,34^ We selected a commercially available di-rhamnolipid (Rha_2_C_10_C_10_), which is intended to serve as a reference biosurfactant for the design of microemulsions and for showing their tunability, as well as their limitations with respect to stability and efficiency. In order to accentuate the biocompatibility and possible applications in cosmetics, isopropyl myristate (IPM) was chosen as oil, while three alkane-1,2-diols were used as hydrophobic co-surfactants. The latter are a class of amphiphiles that are popular co-surfactants^35–37^ and wetting agents^38^ due to their biocompatibility and reported antimicrobial activity.^39–41^ To the best of our knowledge, no such systematic study on the influence of temperature, salinity, pH, and type of co-surfactant on Rha_2_C_10_C_10_-based microemulsions has been reported yet. The first part of this contribution focuses on the influence of these parameters on the phase behavior, followed by a small-angle neutron scattering (SANS) study of the nanostructures formed in selected formulations.

## Experimental Section

In this study we used a technical-grade di-rhamnolipid provided by Evonik Industries AG (Essen, Germany), which is available commercially under the name of Rewoferm RL 100 (batch 99138122) with a molecular mass of *M* ≈ 640 g mol^–1^. It is sold as a 50 wt % solution in H_2_O with a predominant alkyl chain length conformation C_10_C_10_ (>75%) and at a pH of (6.0 ± 0.5). The product contains 10 ppm of the preservative methylchloroisothiazolinone/methylisothiazolinone (CMIT/MIT). As a first step, Rewoferm RL 100 was diluted with bidistilled water (including 10 ppm CMIT/MIT) to a surfactant concentration of 30 wt %. For the preparation of a surfactant analogue with pH = (8.0 ± 0.2) ≫ p*K*_a_, the pH was adjusted with a 30 wt % KOH solution. The surfactant solutions at both pH values, pH = (6.0 ± 0.5) ∼ p*K*_a_ and pH = (8.0 ± 0.2) ≫ p*K*_a_, were then lyophilized at a pressure of 2 × 10^–2^ while being cooled with liquid N_2_. When a water content below 3 wt % was reached, the solid was placed in a vacuum oven at a temperature of 60 °C and a pressure of 10 mbar for 5 days. The residual water content was verified with Karl Fischer titration to be below 1 wt %. Finally, an NMR measurement of the resulting substances in D_2_O was performed, and no sign of degradation could be observed after this drying procedure. Bidistilled water (produced in-house) was used for the phase diagram studies, while all SANS samples were prepared with D_2_O (purity: 99.90%, Euriso-top, Saint-Aubin, France) instead. Potassium chloride (Merck KGaA, Darmstadt, Germany) was added to the aqueous component for selected samples to study the influence of ions. As the oil phase, isopropyl-myristate (C_17_H_34_O_2_, IPM) (purity: ≥ 98%, TCI Chemicals Ltd., Tokyo, Japan) was used. Three different alkane-1,2-diols with differing alkyl chain lengths (*k*) were used as co-surfactants. Octane-1,2-diol (*k* = 8, purity: ≥98%) was purchased from Thermo Fisher Scientific (Waltham, MA, USA). Decane-1,2-diol (*k* = 10, purity: ≥99.0%) and dodecane-1,2-diol (*k* = 12, purity: ≥90%) were purchased from TCI Chemicals Ltd. (Tokyo, Japan). The chemical structure of Rha_2_C_10_C_10_, IPM, and C_k_-1,2-diols is displayed in Figure 1 (right).

**Figure 1:**
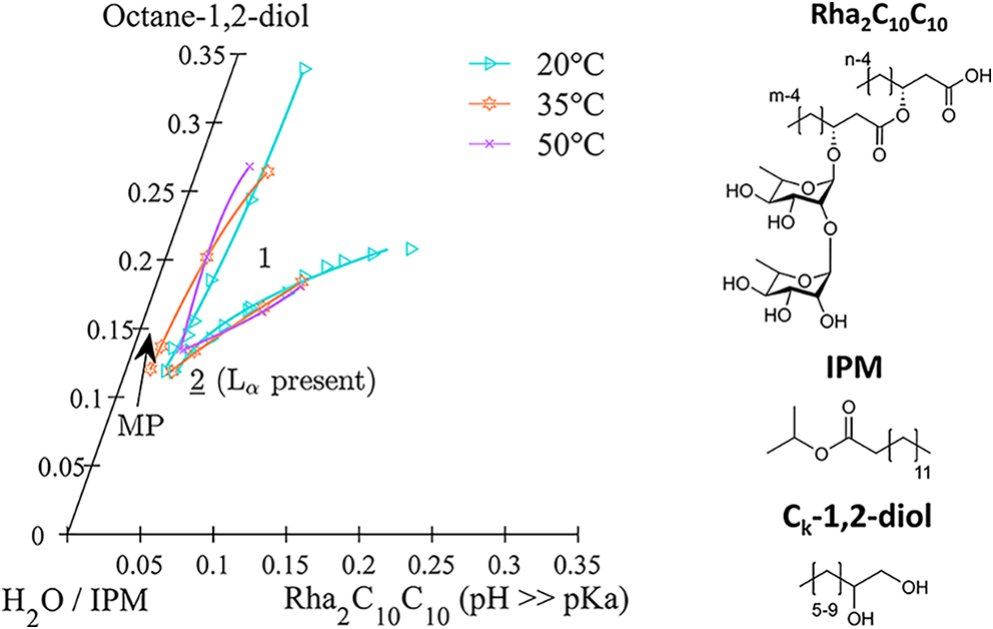
(Left) Phase diagrams of the symmetric (ϕ = 0.50) quaternary microemulsion system H_2_O–IPM–Rha_2_C_10_C_10_ (pH = 8.0 ≫ p*K*_a_)–octane-1,2-diol. The phase diagrams at three different temperatures are displayed, where the one-phase region is labeled with 1. The label 2 (L_α_ present) denotes a coexistence of a water-continuous microemulsion with an oil excess phase and, at low temperatures, with a lamellar LC phase. The label MP denotes a turbid, multiphase region, explained in the main text. (Right) Chemical structure of Rha_2_C_10_C_10_, IPM and alkane-1,2-diol.

### Sample Preparation

Microemulsion samples were prepared by weighing in the desired mass of components (Δ*m* = ± 0.1 mg) into a test tube that was sealed with a Teflon stopper after the addition of a magnetic stirring bar. Each sample was composed of a (pseudo)­component A, comprising water or KCl brine; IPM as the oil component (B); di-rhamnolipid as the surfactant (C); and alkane-1,2-diols as the co-surfactant (D). All mixtures studied were prepared by adjusting equal volumes of IPM and water or KCl brine, i.e.

1 $$\phi =\frac{V_{B}}{V_{A}+V_{B}}=0.50$$

Note that the volumes were calculated using the densities of the (pseudo)­components at the respective temperatures. KCl was chosen as the salt for the brine instead of the more common NaCl to stay consistent with the potassium cation from the KOH base that was used to adjust the pH. The mass fraction of the di-rhamnolipid (C) in the sample is denoted as

2 $$\gamma_{C}=\frac{m_{C}}{m_{A}+m_{B}+m_{C}+m_{D}}$$

while the mass fraction of alkane-1,2-diols (D) is given by

3 $$\gamma_{D}=\frac{m_{D}}{m_{A}+m_{B}+m_{C}+m_{D}}$$

For samples with KCl brine, the salt mass fraction is given by

4 $$\epsilon =\frac{m_{\mathrm{KCl}}}{m_{A}+m_{\mathrm{KCl}}}$$

All samples were then placed in a water bath at *T* = 60 °C for 5 min and stirred to fully solubilize the surfactant and co-surfactant.

### Visual Study

For the phase behavior studies at constant temperature, the samples were placed in a temperature-controlled water bath (Δ*T* = ± 0.1 K). The phase boundaries of the microemulsion systems were determined by titration of either surfactant or co-surfactant and inspection of the sample after each titration step with the stirrer turned off. The number and kind of coexisting phases are determined by visual inspection in both transmitted and scattered light. Birefringence of the occurring lyotropic liquid crystalline phases was detected via crossed linear polarizers. The phase behavior of a quaternary system at a given temperature is usually represented in terms of a section through the phase tetrahedron, resulting in a skewed coordinate system for the resulting pseudoternary phase diagrams. A schematic of this tetrahedron section is depicted in Figure S4 in the Supporting Information.

### Small-Angle Neutron Scattering

Small-angle neutron scattering (SANS) measurements were carried out at the D11 spectrometer at the Institut Laue-Langevin, The European Neutron Source (ILL, Grenoble, France). For all SANS samples, D_2_O or KCl-D_2_O brine was used as the aqueous phase to study the samples in bulk contrast conditions. All samples were prepared as one-phase microemulsions as indicated above, transferred to quartz QX glass cells (Hellma GmbH, Müllheim, Germany) with a path length of 1 mm, and placed in a temperature-controlled copper rack (Δ*T* = ±1 K) provided by the ILL. All samples were inspected prior to and after measurements to exclude the possibility of sample demixing. The neutron beam had a wavelength of λ = 4.6 Å (Δ*λ*/λ = 0.09) with a beam diameter of 14 mm. Measurements were conducted at sample–detector distances of 1.6, 10.2, and 38.9 m with respective collimation distances of 10.2, 10.2, and 35 m. The calibration to absolute intensity units was carried out using direct beam transmission measurements, and data reduction and azimuthal average were performed with the software package GRASP^42^ (v10.24f). The incoherent background was subtracted from all measured curves before subsequent data analysis. The data analysis does not take instrument resolution into account.

### Scattering Theory and Modeling

The peak region of the scattering data was analyzed with the Teubner–Strey model,^43^ developed for bicontinuous microemulsions that models the scattered intensity *I*(*q*) as

5 $$I\left(q\right)=\frac{8\pi \phi \left(1-\phi \right){\left(\Delta \rho \right)}^{2}c_{2}/\xi_{\mathrm{TS}}}{a_{2}+c_{1}q^{2}+c_{2}q^{4}}$$

where Δ*ρ* is the scattering length density contrast and *q* denotes the absolute value of the scattering vector at a total scattering angle of θ, given by *q* = (4π/λ) sin­(θ/2). Eq 5 can be rewritten as *I*(*q*) = 4*Q*/(*πξ*_TS_)/(*a*_2_/*c*_2_ + (*c*_1_/*c*_2_)*q* ^2^ + *q* ^4^), where *Q* = ∫_0_^∞^ *I*(*q*)*q* ^2^ d*q* denotes the Porod invariant of the scattering curve and is equal to 2π^2^ϕ­(1 – ϕ)­(Δ*ρ)* ^2^. The correlation length ξ_TS_ and the periodicity of the water–oil domains, *d*_TS_, can be expressed through the coefficients of the associated Landau–Ginzburg free energy order parameter expansion as

6 $$\xi_{\mathrm{TS}}={\left[\frac{1}{2}{\left(\frac{a_{2}}{c_{2}}\right)}^{1/2}+\frac{1}{4}\frac{c_{1}}{c_{2}}\right]}^{-1/2}$$

and

7 $$d_{\mathrm{TS}}=2\pi {\left[\frac{1}{2}{\left(\frac{a_{2}}{c_{2}}\right)}^{1/2}-\frac{1}{4}\frac{c_{1}}{c_{2}}\right]}^{-1/2}$$

Furthermore, one may also compute the so-called amphiphilicity factor^44^ $f_{a}=c_{1}/\sqrt{4\cdot a_{2}c_{2}}$ , which allows us to quantify the degree of ordering in bicontinuous microemulsions. In general, negative values of *f*_a_ are found for microemulsions, whereas a value of −1.0 corresponds to a long-range, lamellar order. Using eqs 7 and 6, it can be directly related to an effective bending rigidity^45,46^

8 $$\frac{\kappa_{\mathrm{eff}}}{k_{B}T}=\frac{10\pi \sqrt{3}}{64}\;\frac{\xi_{\mathrm{TS}}}{d_{\mathrm{TS}}}=\frac{5\sqrt{3}}{64}\sqrt{\frac{1-f_{a}}{1+f_{a}}}$$

A more detailed investigation combining SANS and neutron spin echo (NSE) measurements revealed that *κ*_eff_ is to be understood as a mixture of the bending modulus κ and the saddle splay modulus κ̅ .^47^

While the Teubner–Strey model is an appropriate choice to model the peak region of the scattering data, deviations can often be observed at high *q* due to the assumption of an infinitely sharp interface between the oil and water phases, resulting in Porod-like scattering (∝*q* ^–4^). In order to account for a finite, blurry interface that stems from the thickness *t* of the interfacial film, we used a modified Porod law^48^ to analyze the high-*q* region

9 $$\lim_{q\to \infty }I\left(q\right)=q^{-4}\;\exp\left(-q^{2}t^{2}\right)\frac{Q}{\pi \phi \left(1-\phi \right)}\left(S/V\right)$$

The specific internal interface (*S*/*V*), obtained from the analysis of the high-*q* region, can be compared to the theoretical value according to the de Gennes–Taupin model for bicontinuous microemulsions that is linked to the width of a single water or oil domain, *d*_TS_/2, as

10 $$d_{\mathrm{TS}}/2=a\frac{\phi \left(1-\phi \right)}{\left(S/V\right)}$$

The geometric proportionality constant *a* has, e.g., been theoretically calculated to be *a* = 6 by de Gennes.^49^ A systematic experimental study by Sottmann et al. on a range of nonionic surfactant-based microemulsion systems led to a value of *a* = 7.16.^50^

## Results and Discussion

In this section, we present the results of our systematic study on the properties of application-relevant microemulsions stabilized by the biosurfactant di-rhamnolipid, aiming to demonstrate their tunability while systematically exploring inherent limitations in terms of stability and efficiency, thereby providing a foundation for rational formulation strategies. Specifically, we are describing the influence of key physical parameters, temperature, pH, and salt content on the phase behavior of symmetric (ϕ = 0.50) microemulsions formed in the quaternary system H_2_O­(KCl brine)–IPM–Rha_2_C_10_C_10_–octane-1,2-diol and the route toward microemulsions with longer chain alkanediols. We present SANS investigations of the nanostructure in the one-phase region close to the so-called optimal point and conclude with observations about the high-*q* region and the specific internal interface (*S*/*V*).

### Phase Behavior

#### Salt-Free Microemulsions

We start our investigation with phase diagram studies of the salt-free microemulsion system (ϵ = 0.000) and at a pH = 8.0 ≫ p*K*_a_, where the rhamnolipid’s carboxylic group is fully deprotonated, rendering it anionic. As is the case for microemulsions formed with the help of closely related alkyl polyglucoside surfactants, temperature is not expected to play a significant role in the tuning of the interfacial curvature that is required for a phase inversion from oil-in-water (O/W) to water-in-oil (W/O) via a bicontinuous state. The reason for this lies in the strong hydrogen bonds formed between the surfactant’s sugar moieties and surrounding water molecules,^8,51,52^ leading to the nearly temperature-invariant phase behavior. Instead, a hydrophobic co-surfactant must be added to the surfactant mixture and takes the role of the effective tuning parameter for the interfacial curvature.^20–22,53^ Nevertheless, phase diagrams representing sections through the phase prism at equal volumes of water and IPM were obtained from Rha_2_C_10_C_10_ and octane-1,2-diol titration experiments conducted at three temperatures in order to investigate the potential influence of temperature on the phase behavior. At all temperatures investigated, a peculiar phase behavior could be observed, as shown in Figure 1 (left). In contrast to closely related microemulsion systems stabilized with alkylpolyglucosides^21,54,55^ no three-phase body, where a microemulsion phase coexists with excess water and excess oil (commonly referred to as ’fish head’), could be observed at any composition or temperature. Instead, the one-phase region at all temperatures has the shape of a funnel. At low co-surfactant fractions, the coexistence of a microemulsion and an oil excess phase (2) governed the phase behavior at high temperatures (*T* ≥ 35 °C). At low temperatures (*T* = 20 °C) the presence of a lamellar phase (L_α_) was observed. Close to the tip of the one-phase funnel, even a three-phase coexistence occurred, comprising a lamellar phase (L_α_), a microemulsion and an oil excess phase.

At low rhamnolipid concentrations, a turbid, multiphase (MP) mixture was present at all temperatures, which did not phase-separate over the course of a few weeks. Furthermore, the upper phase boundary runs almost parallel to the co-surfactant axis, showcasing the ineffectiveness of octane-1,2-diol to invert the curvature of the interfacial film. This funnel-like phase behavior is strikingly reminiscent of that of the widely studied ionic surfactant dioctyl sodium sulfosuccinate (AOT). It has been shown that microemulsions formed with this double-chain ionic surfactant require the addition of a salt to observe the three-phase ’fish head’ and a balanced microemulsion with a minimum interfacial tension. Kahlweit et al.^56^ systematically investigated the influence of salt content ϵ on the phase diagrams of temperature-controlled microemulsions formed with ionic surfactants. They concluded that this lack of a three-phase body in the salt-free system can be ascribed to the presence of a connected critical line that runs from the water-rich to the oil-rich side of the Gibbs phase prism. In their temperature-controlled brine–oil–ionic surfactant systems, the ’breaking’ of this critical line could be achieved either by increasing the hydrophobicity of the oil phase or by tuning the chemical potential of the aqueous phase by adding an appropriate lyotropic salt. Furthermore, the presence of salt pushes back the lamellar phase in AOT-based microemulsions,^57^ an effect that is directly coupled to the changes of the bending modulus (κ) and the saddle-splay modulus (κ̅) of the interfacial film. Typically, the addition of salt leads to a decrease of κ and an increase in κ̅, resulting in a destabilization of the lamellar phase and a preferred formation of connected, bicontinuous structures.^58,59^

We conclude this section by pointing out that a qualitatively identical, funnel-like phase behavior was observed for salt-free microemulsions formulated at pH ∼ p*K*_a_ (not shown). For both pH conditions, no three-phase body could be observed in salt-free formulations (ϵ = 0.000). Finally, attempts to replace the co-surfactant octane-1,2-diol (alkyl chain length *k* = 8) with longer chain analogues (*k* = 10 and 12 respectively) to increase the efficiency of the amphiphile mixture led to the formation of highly viscous, turbid mixtures containing anisotropic LC phases. We ascribe this effect to the ionic character of rhamnolipid that, together with the sterically big sugar headgroup, hinders an inversion of the amphiphilic film.

#### Effect of Salinity

Following the approach of Kahlweit et al.,^56^ who demonstrated that the addition of salt to salt-free AOT microemulsions (ϵ = 0.000) induces the formation of a three-phase body by reducing electrostatic repulsion, KCl was added to the rhamnolipid microemulsion, and the resulting phase behavior was subsequently investigated. We used the di-rhamnolipid at pH = 8.0 ≫ p*K*_a_ in order to have the surfactant molecules in their deprotonated ionic state and recorded the phase diagrams at different KCl concentrations ϵ in the aqueous phase. We chose a temperature of 35 °C to avoid the possible occurrence of LC phases at low salt concentrations and temperatures.

As can be seen in Figure 2 (left), the phase behavior drastically changes upon addition of KCl to water (ϵ = 0.010). Compared to the salt-free formulation, the phase boundaries shift to higher rhamnolipid concentrations, as the ionic interaction between the charged head groups of neighboring amphiphilic films is screened, leading to a partial loss of solubilization capacity. As a consequence of this ’salting out’ effect,^56,60^ the surfactant rhamnolipid becomes effectively more hydrophobic, as also demonstrated by a shift of the phase boundaries to lower co-surfactant concentrations. This shift is best described by the coordinates of the *X̃*-point, that denote the respective minimum amount of surfactant (γ̃_C_) and co-surfactant (γ̃_D_) required for the formation of a one-phase microemulsion and is commonly used to quantify the efficiency of a microemulsion formulation. They were determined by the intersection of the second-order polynomial fits of the respective upper and lower phase boundaries. For ϵ = 0.000, the *X̃*-point is located at (γ̃_C_ = 0.025, γ̃_D_ = 0.110), though it is not a true *X̃*-point due to the absence of a three-phase region, and shifts to (γ̃_C_ = 0.095, γ̃_D_ = 0.160) at a salt concentration of ϵ = 0.010. The latter represents a true *X̃*-point, as the addition of a small amount of salt induces the formation of a three-phase body, consistent with the observations of Kahlweit et al. in the AOT system.^56^ Furthermore, now the addition of octane-1,2-diol enables the formation of a 2̅ region at high octane-1,2-diol contents, in which an oil-continuous microemulsion coexists with an aqueous excess phase. This ability to invert the amphiphilic film curvature upon addition of octane-1,2-diol can be understood as a consequence of the reduced headgroup repulsion of neighboring rhamnolipid molecules due to screened electrostatic interactions.

**Figure 2:**
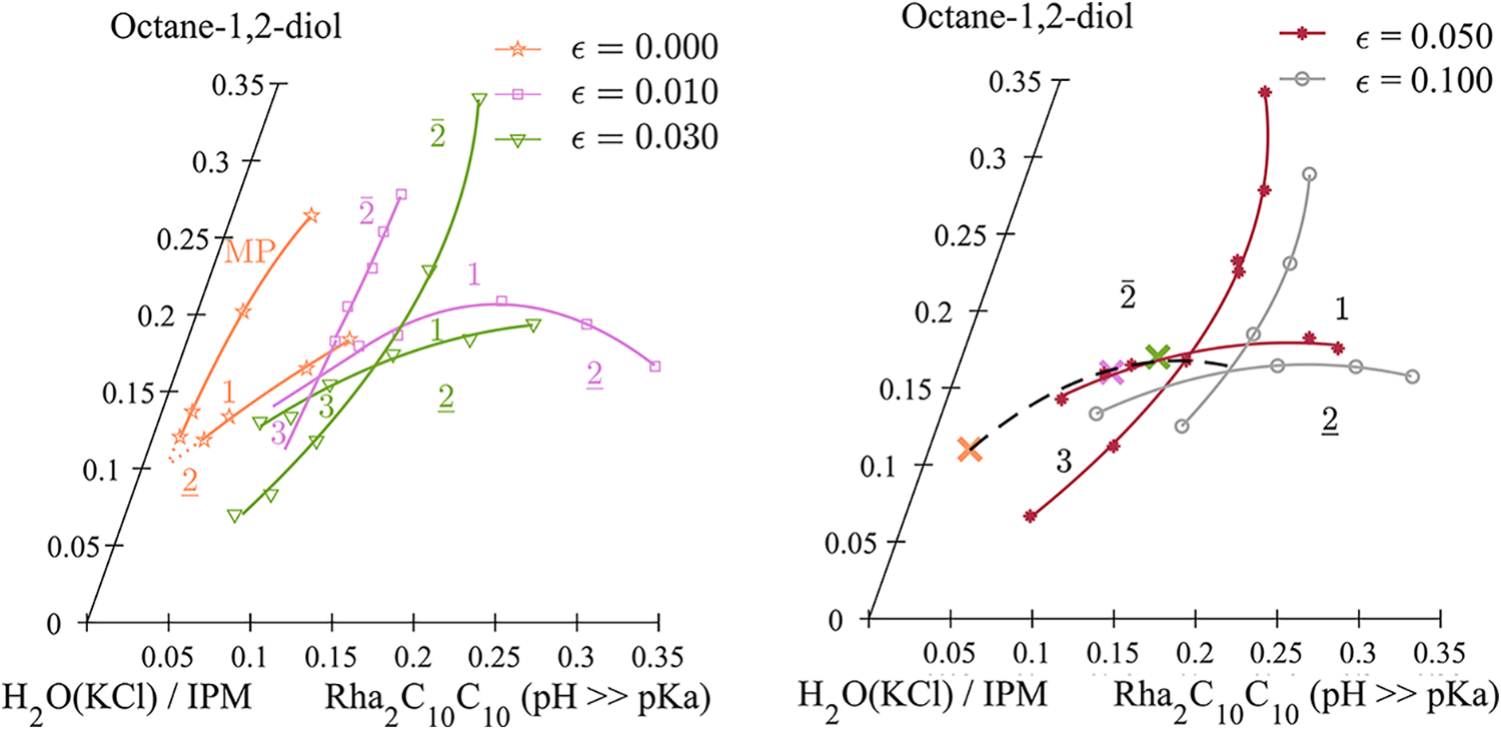
Phase diagrams of symmetric microemulsions (ϕ = 0.50) formed in the quaternary system H_2_O­(KCl)–IPM–Rha_2_C_10_C_10_ (pH = 8.0 ≫ p*K*_a_)–octane-1,2-diol at varying salt concentrations ϵ. On the left, the phase diagrams at low salt concentrations ϵ are displayed. On the right, phase diagrams for the two highest salt concentrations are displayed. The crosses indicate the location of the (pseudo-) *X̃*-points of the phase diagrams measured at low salt concentrations and are connected by a dashed line to visualize the trajectory. All phase diagrams were measured at a temperature of 35 °C.

Increasing the salt concentration further (ϵ = 0.030), this pattern continues, as the phase boundaries shift to even higher surfactant concentrations and the *X̃*-point is observed at (γ̃_C_ = 0.120, γ̃_D_ = 0.170). At the highest salt concentrations studied (ϵ = 0.050 and 0.100), the high amounts of salt in the aqueous phase lead to a further loss in solubilization efficiency. Comparing the two phase diagrams in Figure 2 (right), it is evident that the phase boundaries also shift toward lower octane-1,2-diol concentrations, as the rhamnolipid becomes increasingly hydrophobic with rising salt content. The coordinates of the *X̃*-points at all salt concentrations are summarized in Tab. S1 in the Supporting Information. For all further studies, we chose a constant salt concentration of ϵ = 0.030, in order to preserve characteristic microemulsion phase behavior and a reasonable solubilization efficiency.

While the addition of salt led to the aforementioned characteristic microemulsion behavior and the formation of a three-phase region, the surfactant mixture is still quite hydrophilic, as shown by the high amounts of co-surfactant that are needed to form a microemulsion (1). Attempts to use longer chain alkanediols as co-surfactants again led to the formation of viscous LC phases at a pH = 8.0 ≫ p*K*_a_. Therefore, as a next step, we investigated the influence of the pH on the phase behavior with the goal to enable more efficient formulations with longer-chain co-surfactants.

#### Effect of pH and Co-surfactant Chain Length

Compared to pH = 8.0 ≫ p*K*_a_, where the Rha_2_C_10_C_10_ is fully deprotonated and therefore behaves as an anionic, strongly hydrophilic surfactant, lowering the rhamnolipid’s pH to pH = 6.0 ∼ p*K*_a_ will result in a surfactant mixture, containing roughly equal amounts of protonated and deprotonated rhamnolipid molecules in the interfacial film. The corresponding impact on the phase behavior is shown in Figure 3 (left), where the phase diagrams at both pH values are compared at a fixed salt concentration ϵ = 0.030 and a temperature of *T* = 35 °C. Lowering the pH of Rha_2_C_10_C_10_ to pH ∼ p*K*_a_, strongly changes the phase behavior. The increased hydrophobicity of the partially protonated Rha_2_C_10_C_10_ shifts the phase boundaries to lower co-surfactant concentrations. In contrast to studies that predict synergistic effects in mixtures of ionic and nonionic surfactants,^61,62^ the solubilization efficiency of our surfactant mixture at pH ∼ p*K*_a_ is decreased, with the *X̃*-point located at (γ̃_*C*_ = 0.155, γ̃_*D*_ = 0.130). This behavior partially stems from the higher monomeric solubility of the protonated Rha_2_C_10_C_10_ in IPM, but it can also be discussed in terms of the almost unchanged bending rigidity of the amphiphilic film-a point that we will revisit further below in the discussion of the SANS curves.

**Figure 3:**
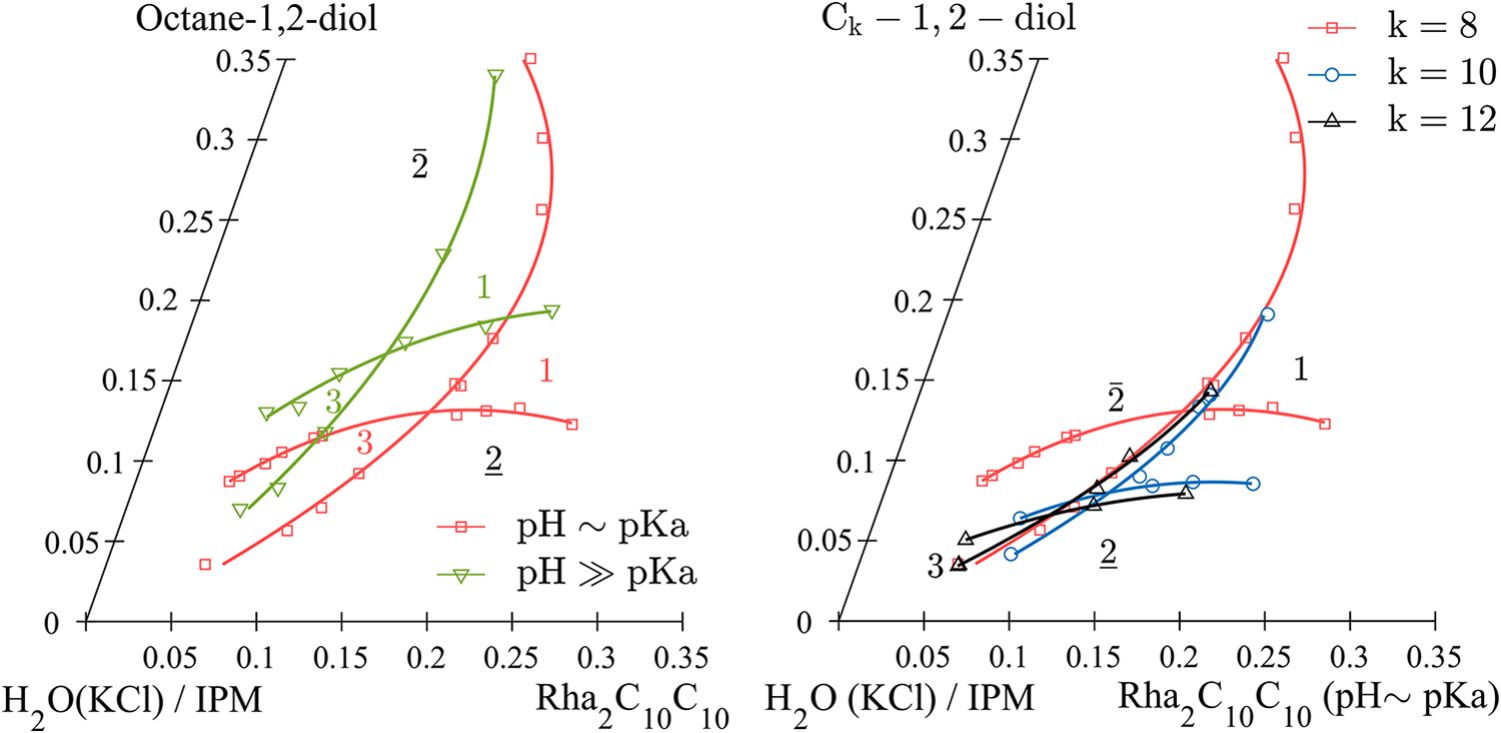
Phase diagrams of symmetric microemulsions (ϕ = 0.50) formed in the quaternary system H_2_O­(KCl) (ϵ = 0.030)–IPM–Rha_2_C_10_C_10_–C_k_-1,2-diol. On the left, the phase diagrams of the system with the co-surfactant octane-1,2-diol (*k* = 8) are displayed at pH = 8.0 ≫ p*K*_a_ and pH ∼ p*K*_a_ for comparison. The right figure shows phase diagrams at pH ∼ p*K*_a_ for varying co-surfactant alkyl chain lengths (*k* = 8, 10, and 12). All phase diagrams were measured at a temperature of 35 °C.

The lowering of the 2 – 1 phase boundary to lower co-surfactant concentrations reflects the more hydrophobic nature of the amphiphilic film, where the protonated rhamnolipid molecules act as a third, more hydrophobic pseudocomponent in the amphiphile mixture. Notably, neglecting the high monomeric solubility of octane-1,2-diol in IPM (see below), the total amount of surfactant at the *X̃*-point (γ̃ = γ̃_C_ + γ̃_D_) stays almost constant with γ̃(pH ≫ p*K*_a_) = 0.290 and γ̃(pH ∼ p*K*_a_) = 0.285, indicating that the total efficiency of the surfactant mixture hardly changes at lower pH.

Furthermore, the more hydrophobic amphiphilic film formed at pH ∼ p*K*_a_ allowed the use of longer-chain alkanediols for the formulation of microemulsions with improved solubilization of brine and IPM. The recorded phase diagrams are shown in Figure 3 (right). Starting from the previously discussed phase diagram with octane-1,2-diol (*k* = 8) as a co-surfactant, the use of decane-1,2-diol (*k* = 10) strongly shifts the 2 – 1 phase boundary to lower co-surfactant fractions with the *X̃*-point located at (γ̃_C_ = 0.135, γ̃_D_ = 0.080), reflecting a marked increase in solubilization efficiency of the amphiphile mixture to γ̃(*k* = 10) = 0.215. Using dodecane-1,2-diol (*k* = 12), the 2 – 1 phase boundary shifts further toward lower co-surfactant fractions. Accordingly, the *X̃*-point is located at (γ̃_C_ = 0.105, γ̃_D_ = 0.065), corresponding to minimum amphiphile concentration for complete solubilization of brine and oil, γ̃ = 0.170. The smaller shift of the 2 – 1 phase boundary observed for the dodecane-1,2-diol system may result from the higher monomeric solubility of dodecane-1,2-diol in IPM, leading to a reduced partitioning into the interfacial film.^63^

We want to conclude this section with a discussion of the shape and position of the phase boundaries and their relation to their respective ternary subsystems. The downward shift of the lower 2 – 1 phase boundary (see Figure 3) can be explained by the effect of the alkanediol chain length on the ternary BCD subsystems of the studied quaternary microemulsion H_2_O (A)–IPM (B)–Rha_2_C_10_C_10_ (C)–alkane-1,2-diol (D) (see ref 21 and Figure 3 and 4 therein). As the co-surfactant chain length increases, the miscibility gap between the IPM and rhamnolipid in the BCD system is expected to shrink, since alkanediols function as both a co-surfactant and cosolvent. The latter effect increases the oil polarity and its ability to mix with the rhamnolipid, which together cause the observed shift of the lower phase boundary. In contrast, the upper phase boundary remains nearly unchanged for all co-surfactant alkyl chain lengths *k* studied within the measured region. This insensitivity of the upper phase boundary might be related to compensating effects: On the one hand, the miscibility gap of the ternary subsystem ACD is expected to increase with increasing alkanediol chain length. On the other hand, the increased monomeric solubility of longer chain alkanediols in IPM leads to their preferential partitioning into the oil phase.

**Figure 4:**
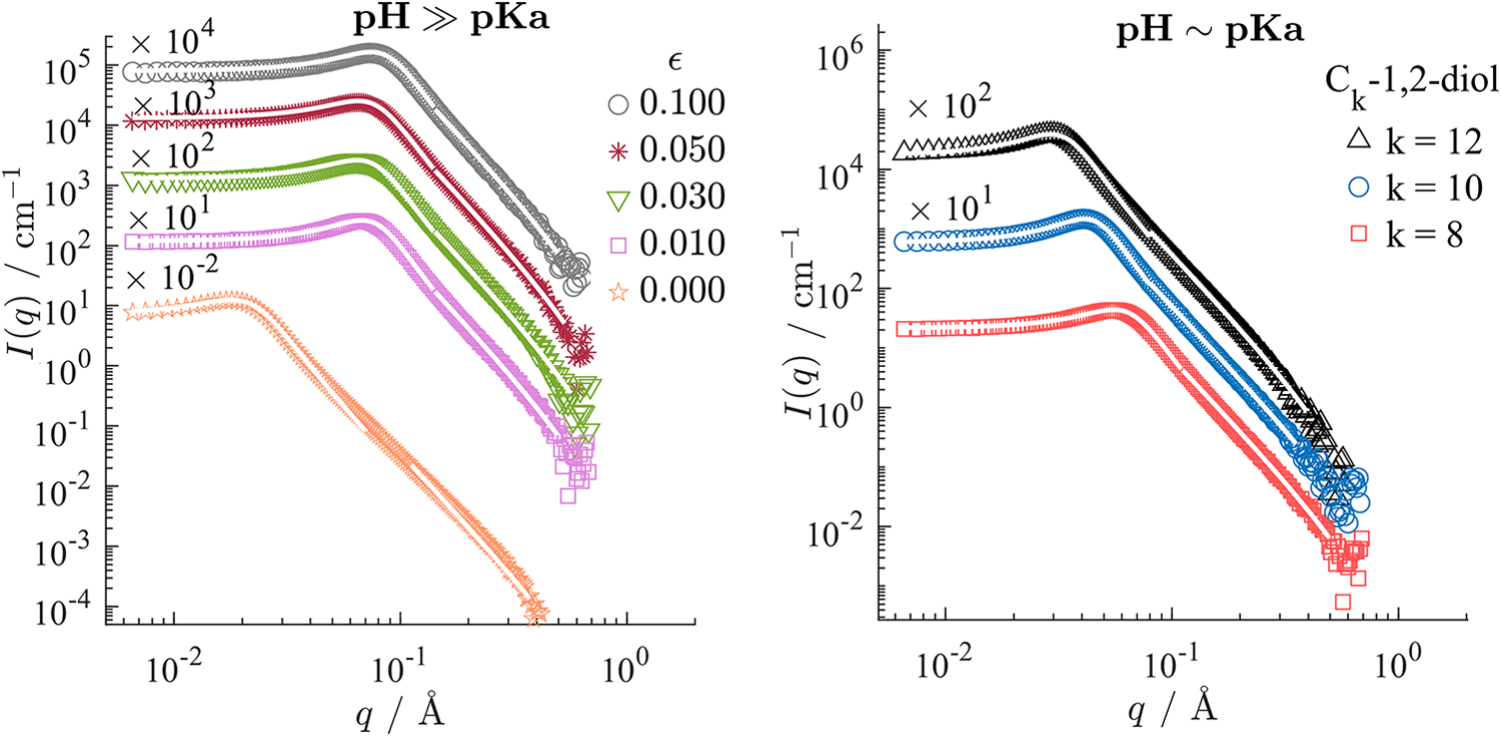
Bulk contrast SANS data of symmetric microemulsions (ϕ = 0.50) formed by D_2_O­(KCl)–IPM–Rha_2_C_10_C_10_–C_k_-1,2-diol recorded close to the respective *X̃*-point at 35 °C. On the left, SANS curves of microemulsions at pH ≫ p*K*_a_ formulated with the co-surfactant C_8_-1,2-diol and varying salt concentration ϵ are shown. On the right, SANS data of microemulsions at pH ∼ p*K*_a_ and ϵ = 0.030 for differing co-surfactant alkyl chain lengths *k* are displayed. Measured intensities were multiplied by arbitrary factors for ease of visualization. Model fits, performed using a combination of the Teubner–Strey model (eq 5) for the peak region and a modified Porod law (eq 9) for the high-*q* regime, are shown as white lines.

### Small-Angle Neutron Scattering

In the first part of this study, we demonstrated that salt addition strongly affects the phase behavior of the investigated symmetric di-rhamnolipid microemulsions. To study the impact of salt, pH, and co-surfactant chain length on the microemulsion structure, SANS curves were recorded close to the respective *X̃*-point at 35 °C. Bulk contrast was achieved by using D_2_O instead of H_2_O. The SANS samples’ compositions were selected to match the surfactant and co-surfactant molarities of the corresponding hydrogenated systems, while maintaining equal volume fractions of D_2_O­(KCl) and IPM (ϕ = 0.50). The composition of all samples is summarized in Tab. S4 to S6 in the Supporting Information.

The SANS data for microemulsions at pH ≫ p*K*_a_, formulated with the co-surfactant octane-1,2-diol for salt concentrations ϵ between 0.000 and 0.100 are shown in Figure 4 (left). All curves show the typical scattering behavior of symmetrical microemulsions close to the *X̃*-point in bulk contrast. At low *q*, a plateau of constant scattering intensity is observed for all samples, followed by a broad structure factor peak at mid-*q* that reflects the periodicity of the water (and oil) domains in the microemulsion. At higher scattering vectors, the intensity decays according to Porod’s law (∝ *q* ^–4^), as the scattering from the interface between the domains dominates in this *q*-region. At the highest *q*, the scattering intensity decays more strongly than predicted by ideal Porod scattering, which is typically attributed to a smeared rather than sharp scattering length density profile caused by hydration, penetration, and molecular motion. All SANS spectra were analyzed with a combination of the Teubner–Strey model (eq 5) for the peak region and a modified Porod law (eq 9) for the high-*q* data. While the Teubner–Strey (TS) model gives values for the typical domain size of water and oil channels, *d*_TS_/2, and the correlation length ξ_TS_, the analysis of the high-*q* region allows for the determination of the specific internal interface (*S*/*V*), taking into account the scattering invariant *Q*. For ϵ = 0.000, the structure factor peak is located at the lowest *q*-value, reflecting the formation of large structures, as the salt-free system requires the lowest surfactant concentration to form a one-phase microemulsion (see eq 10), which might be explained by an additional stabilization from electrostatic repulsion. A fit to the TS-model gives a typical size of water (and oil) domains as *d*_TS_/2 = 156 Å and a correlation length ξ_TS_ = 108 Å. The amphiphilicity factor *f*_a_ = −0.65 indicates a moderately well-structured microemulsion.^44^ Interestingly, we found that increasing the fraction of octane-1,2-diol in the amphiphile mixture at a slightly lower total amphiphile concentration, and thus approaching the upper phase boundary (see Figure 1, left), the scattering peak becomes less pronounced and moves to lower *q*. As shown in more detail in the Supporting Information (Figure S6 and Table S8), the amphiphilicity factor increases from values around −0.65 to ca. −0.09, indicating a loss of order in the microemulsion structure^44^ close to the upper phase boundary, where the amphiphilic film is largely composed of octane-1,2-diol. A similar behavior was observed for microemulsions stabilized by short-chain nonionic surfactant mixtures.^64^

Note that, while the peak region of the recorded SANS curves can be quantitatively described by the Teubner–Strey model, systematic deviations from the modified Porod law can be observed at high *q* for the salt-free (ϵ = 0.000) microemulsions, a phenomenon that will be discussed further below.

Upon increasing the salt concentration to ϵ = 0.010, the structure factor peak shifts to higher *q*, corresponding to a significantly reduced domain size, *d*_TS_/2 = 43 Å. In addition, the amphiphilicity factor becomes more negative, reaching −0.74, indicating a higher degree of order in the microemulsion. The decrease of the domain size in comparison with the salt-free formulation reflects a reduction of the rhamnolipid’s efficiency in solubilizing equal amounts of water and IPM, as the electrostatic interactions between the charged head groups at opposite sides of water domains are partially screened after the addition of salt. A further increase in salinity up to a concentration of ϵ = 0.100 has a comparatively small influence on the size of the water (and oil) domains, which decreases only slightly to *d*_TS_/2 = 39 Å for the highest salt concentration measured. Also, the values of *f*_a_ remain consistently around −0.7, confirming the presence of well-structured microemulsions independent of the salinity. The comparatively weak influence of salt addition at salinities ϵ > 0.010 can be understood as a consequence of the fact that the electrostatic interactions are largely screened. Examining the high-*q* regime of the scattering curves, it is particularly interesting that for ϵ ≥ 0.010, the decay of all SANS curves is better described by the modified Porod law, (eq 9) with a thickness *t* = 1.5 Å of the blurry interface, as shown in more detail in Figure S5 for one exemplary scattering curve. Clear deviations from this behavior are observed for the SANS curve of the salt-free sample (ϵ = 0.000), as revisited further below. For all other SANS curves, the value found for the thickness *t* is rather low when compared with, e.g., microemulsions formulated with polyethylene glycol alkyl ethers, where values of *t* = 4–5 Å are usually reported.^46^ However, a SANS study on microemulsions formulated with an alkyl monoglucoside and a medium-chain alcohol, an amphiphile combination comparable to ours, also reported a quite low value of *t* = 1 Å.^65^ Given the even bigger, hydrated double rhamnose headgroup of Rha_2_C_10_C_10_, the physical meaning of the obtained values of *t* should be treated with caution and might not reflect the ’true’ interfacial roughness. Instead, the surprisingly low values of *t* could stem from the contribution of the hydrated sugar head groups to the local scattering length density (SLD) profile. Such a three-level SLD profile would lead to additional, nontrivial terms in the asymptotic high-*q* behavior^66–68^ that could explain the observed low values of *t* for microemulsions formulated with sugar surfactants. The fit parameters obtained from the analysis with the TS-model, as well as the specific internal interface (*S*/*V*) obtained from the Porod analysis, are summarized in Table 1.

**Table 1:** Fit Parameters from the Analysis of the SANS Curves Shown in Figure 4 (Left)^a^

ϵ	*d*_TS_/2/Å	ξ_TS_/Å	*Q*/cm^–1^ Å^–3^	(*S*/*V*)/Å^–1^	*t*/Å	*f*_a_	κ_eff_/*k*_B_ *T*
0.000	156	108	0.0165	0.0169	1.5	–0.65	0.29
0.010	43	35	0.0119	0.0482	1.5	–0.74	0.35
0.030	45	35	0.0116	0.0491	1.5	–0.71	0.33
0.050	44	33	0.0116	0.0513	1.5	–0.69	0.32
0.100	39	30	0.0104	0.0586	1.5	–0.71	0.33

a Shown are the domain size, *d*_*T*S_/2, and correlation length, ξ_TS_, from the TS-model along with the invariant *Q* calculated from the second moment of the scattering intensity and the specific internal interface from the Porod analysis, (*S*/*V*), including the width of the blurry interface *t*. The amphiphilicity factor is denoted by *f*_a_, and the temperature-normalized effective bending rigidity is denoted by κ_eff_/*k*_B_ *T*.

Also, the influence of pH and co-surfactant chain length on the microstructure is studied by means of SANS measurements of microemulsions at pH ∼ p*K*_a_, formulated at a salt concentration ϵ = 0.030, using octane-1,2-diol (*k* = 8), decane-1,2-diol (*k* = 10), and dodecane-1,2-diol (*k* = 12) as co-surfactant, as shown in Figure 4 (right). At a fixed co-surfactant chain length *k* = 8, lowering the pH to pH ∼ p*K*_a_ does not change the structure of the microemulsions significantly. A slight increase in the domain size *d*_TS_/2 from 45 to 52 Å when compared with the microemulsion formulated at pH ≫ p*K*_a_ can be explained by the fact that the latter sample was prepared at a slightly lower total amphiphile concentration, closer to the *X̃*-point (see sample composition in the SI). The nearly constant effective bending rigidity κ_eff_ = 0.33 *k*_B_ *T* for both pH values indicates that there is no significant influence of the degree of deprotonation of Rha_2_C_10_C_10_ on the flexibility of the interfacial film. Similarly, Farago, Gradzielski, and co-workers^69^ found only a negligible influence of the charge density of mixed nonionic–ionic interfacial films on the bending elasticity of the latter, using a combination of SANS and NSE. They argued that the increased repulsion between head groups leads to larger areas available for both the head groups and the alkyl chains, resulting in a softening of the amphiphilic film that counterbalances the simple electrostatic contribution.

Considering now the variation of the SANS data with increasing co-surfactant chain length *k*, it becomes apparent that the structure factor peak shifts to lower *q*. This trend demonstrates that microemulsions formulated with a longer chain co-surfactant are able to form larger structures due to the lower amphiphile concentration required for the solubilization of water and IPM (see Figure 3 (right)). Thus, while a domain size *d*_TS_/2 = 52 Å was found for *k* = 8, an increase to 72 Å and finally 99 Å was observed for *k* = 10 and *k* = 12, respectively. Despite this systematic trend in domain size with co-surfactant chain length, the amphiphilicity factor *f*_a_ remains consistently ≈ – 0.7, leading to an effective bending rigidity κ_eff_/*k*_B_ *T* ≈ 0.3 (see eq 8). This is attributed to the fact that, although the longer co-surfactant chains increase the bare bending rigidity of the mixed amphiphilic film, this effect is compensated by enhanced thermal fluctuations at larger domain sizes,^70–72^ so that the degree of order close to the *X̃*-point remains unchanged. Note that, interestingly, at pH ∼ p*K*_a_, the high-*q* decay of all SANS curves is almost quantitatively described by the modified Porod law. All fit parameters obtained from the analysis of the SANS curves are summarized in Table 2.

**Table 2:** Fit Parameters from the Analysis of the SANS Curves Shown in Figure 4 (Right)^a^

*k*	*d*_TS_/2/Å	ξ_TS_/Å	*Q*/cm^–1^ Å^–3^	(*S*/*V*)/Å^–1^	*t*/Å	*f*_a_	κ_eff_/*k*_B_ *T*
8	52	40	0.0127	0.0419	1.5	–0.71	0.33
10	72	64	0.0147	0.0273	1.5	–0.77	0.38
12	99	82	0.0155	0.0194	2.0	–0.74	0.35

a Shown are the domain size, *d*_TS_/2, and correlation length, *ξ*_TS_, from the TS-model along with the invariant *Q* and the specific internal interface from the Porod analysis, (*S*/*V*), including the width of the blurry interface, *t*. The amphiphilicity factor is denoted by *f*_a_, and the temperature-normalized effective bending rigidity is denoted by *κ*_eff_/*k*_B_ *T*.

Returning to the point made above, particularly, the SANS curve of the salt-free (ϵ = 0.000) microemulsion at pH ≫ p*K*_a_ (Figure 4) (left) shows clear deviations from the modified Porod law in the high-*q* regime. To investigate this surprising observation in more detail, the SANS data were alternatively represented in a modified Porod plot, as shown in Figure 5. When plotting *q* ^4^exp­(*q* ^2^ *t* ^2^)*I*(*q*) vs *q*, bulk contrast SANS measurements of microemulsions are expected to exhibit a maximum (structure factor peak) followed by a constant plateau in the Porod region at high *q*, where the scattering from the diffuse interface dominates. While all Porod plots in Figure 5 (right) recorded at pH ∼ p*K*_a_ and ϵ = 0.030 clearly follow the expected behavior, all data recorded at pH ≫ p*K*_a_, shown in Figure 4 (left), exhibit an increasing trend at high *q* instead of the expected plateau.

**Figure 5:**
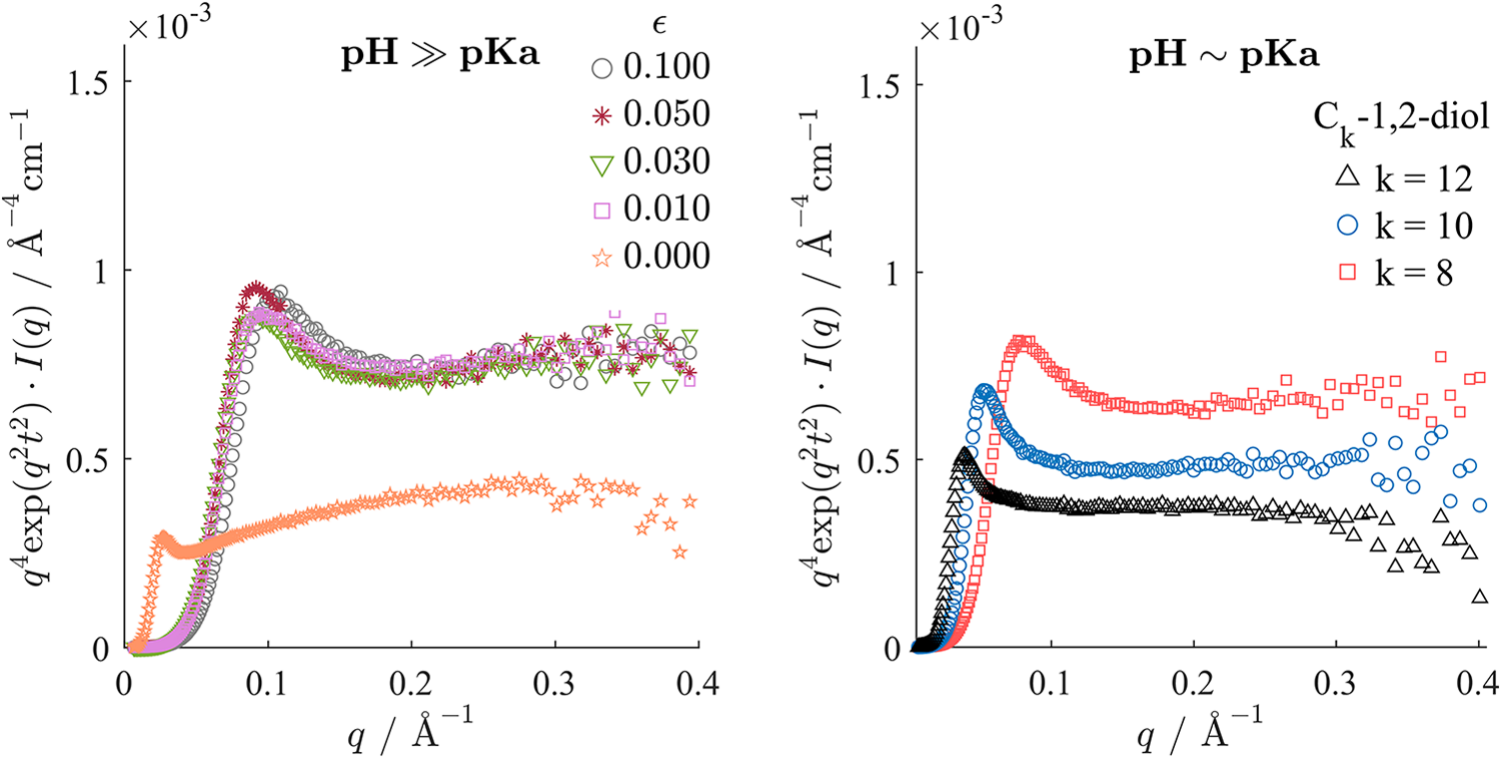
Modified Porod plots of the SANS data shown in Figure 4, corrected for the blurry interface of thickness *t*. On the left, Porod plots of the salt variation at pH ≫ p*K*_a_, shown in Figure 4 (left), are displayed. On the right, Porod plots for the co-surfactant chain length variation at pH ∼ p*K*_a_, shown in Figure 4 (right), are displayed.

Particularly for the salt-free sample, a broad shoulder can be observed that spans the whole high-*q* region and therefore clearly deviates from the expected Porod scattering. Traces of this additional signal can also be observed at the highest *q* for all other samples studied at pH ≫ p*K*_a_. This additional scattering contribution greatly influences the Porod region, leading to an overestimation of the extracted specific internal interface (*S*/*V*), as shown in Figure 6, which displays a double-logarithmic plot of *d*_TS_ against (*S*/*V*). According to geometric models of Debye et al., Talmon and Prager^73^ and de Gennes and Taupin (eq 10), those two quantities should be inversely proportional, taking into account the volume fraction term ϕ­(1 – ϕ) and a geometric proportionality constant *a*. While values between 6 and 7 have been reported in past contributions,^49,50^ we find *a* = 10.6 for the microemulsions formulated with Rha_2_C_10_C_10_. One might assume that the larger value of *a* is mainly due to the aforementioned additional scattering contribution at high *q* and contributions from multiple scattering (see below). However, the salt-free sample (ϵ = 0.000), in which both contributions are particularly pronounced and which clearly deviates from the inverse proportionality observed for the other samples, was excluded from the determination of the geometric constant *a*. We rather propose that the high value of *a* is due to an additional film-contrast contribution arising from the rhamnolipid’s protonated double-rhamnose headgroup, which leads to nontrivial terms (∝ *q* ^–2^) in the asymptotic high-*q* behavior^66–68^ (see also the discussion above in the context of the observed low values of *t* in the Gaussian term (eq 9) for sugar surfactants). As this contribution becomes more pronounced in microemulsions with larger periodicity, where the bulk scattering has already decayed most strongly, larger values of *S*/*V* are particularly determined in these samples, leading to a larger slope of the *d*_TS_ versus (S/V) plot.

**Figure 6:**
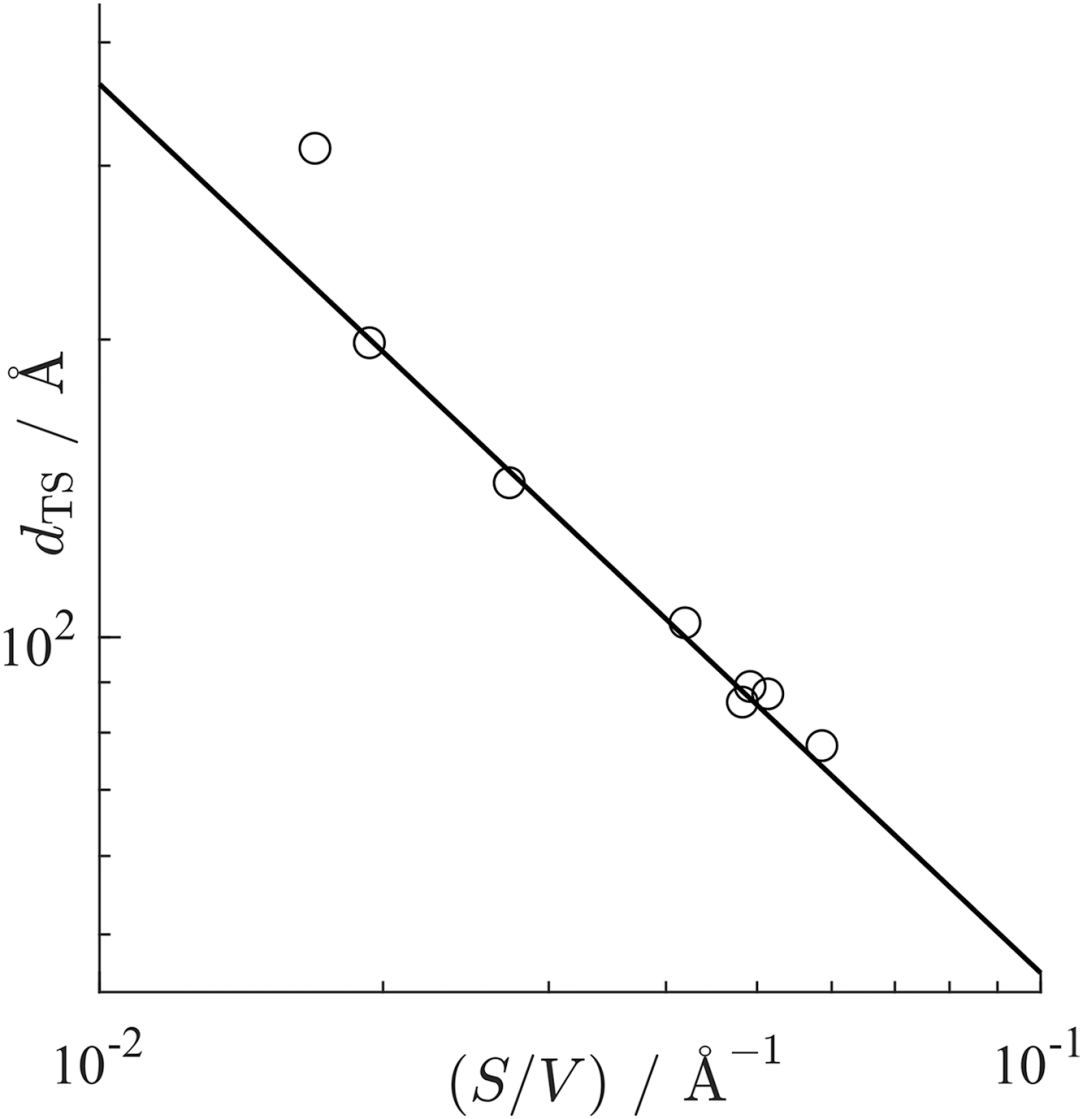
Double-logarithmic plot of the periodicity *d*_TS_ against the specific internal interface (*S*/*V*) for all measured SANS samples, as extracted from the respective fits to the low- and high-*q* regions in Figure 4. The power-law fit line was calculated without the data point for the salt-free sample (ϵ = 0.000) at the lowest (*S*/*V*) and is discussed in the main text.

As of today, we can only speculate on the origin of the scattering feature at high *q* observed for the microemulsions at pH ≫ p*K*_a_, where the di-rhamnolipid is deprotonated, giving rise to ionic interactions. However, we would like to briefly comment on these surprising observations in the SANS data: (1) In order to investigate the possible influence of multiple scattering, the SANS data of the salt-free sample (ϵ = 0.000) was treated with the MuScatt algorithm^74,75^ that allows to mitigate the impact of multiple scattering. The result is shown and compared with the original data in Figure S3 in the Supporting Information. The removal of multiple scattering effects with the MuScatt method led to a slight sharpening of the correlation peak, reflected in an increase of the correlation length ξ_TS_ from 108 to 131 Å, accompanied by a decrease of the amphiphilicity factor *f*_a_ from −0.65 to −0.74 when compared with the original data. The latter value indicates a well-structured microemulsion and is in line with the *f*_a_ values found for all other samples, as discussed above. However, the scattering feature at high *q* was not removed by the algorithm and cannot be explained as a multiple scattering effect.

(2) The additional scattering feature is only visible at large wave vectors *q*, its origin is therefore indubitably to be found on the inter- or intramolecular length scale. As all SANS measurements were conducted in bulk contrast with D_2_O as the aqueous and h-IPM as the oil phase, the contrast contribution of the rhamnolipid’s protonated alkyl chains is deemed negligible in comparison to that of the protonated double rhamnose headgroup. Recent molecular dynamics studies^76^ have shown that the headgroup of Rha_2_C_10_C_10_ tends to form a ’shielding pocket’, i.e., a closed conformation of the rhamnose rings in order to shield the charge of the deprotonated carboxylic unit.^77^ The rhamnolipid’s headgroup conformation is thus highly dependent on pH and the ionic strength of the solvent, which could explain the observed strong dependency of the molecular scattering feature on both salinity and pH. A second possible origin of this scattering feature could lie in the interfacial segregation of Rha_2_C_10_C_10_ due to electrostatic repulsion between neighboring rhamnolipid molecules.

## Conclusions

In this contribution, we have investigated symmetric microemulsions of type H_2_O (brine)–isopropyl myristate–Rha_2_C_10_C_10_–alkane-1,2-diol that are relevant in, e.g., cosmetic applications due to the components’ biocompatibility. We present a systematic study, combining classic phase diagram measurements in combination with small-angle neutron scattering (SANS), showcasing the influence of all major formulation parameters on the phase behavior and nanostructure of symmetric microemulsions, i.e., those containing equal amounts of brine and oil. In analogy to alkyl polyglucoside sugar surfactants (C_m_G_n_), temperature is found to play hardly any role in the phase behavior and microstructure of the studied di-rhamnolipid microemulsion, as the large hydrophilic sugar headgroup is hardly dehydrated at high temperatures. For formulations without added salt (ϵ = 0.000), the isothermal addition of a hydrophobic co-surfactant of type octane-1,2-diol did not lead to an inversion of the amphiphilic film curvature from O/W to W/O. Instead of a Kahlweit fish with a three-phase region, only a funnel-shaped one-phase region was observed, a phase behavior that is strikingly similar to that observed for salt-free microemulsions formed by the ionic double-chain surfactant AOT. In particular, at pH ≫ p*K*_a_, Rha_2_C_10_C_10_ behaves like an anionic surfactant, so that, analogously to AOT microemulsions, it was possible, by the addition of KCl and screening of ionic interactions, to invert the curvature of the amphiphilic film via the addition of octane-1,2-diol. The addition of salt led to a decrease in solubilization efficiency of the amphiphile mixture, as the minimum amount of amphiphiles needed to solubilize equal amounts of oil and water rose strongly from γ̃(ϵ = 0.000) = 0.135 to γ̃(ϵ = 0.100) = 0.325, as the ionic contribution of Rha_2_C_10_C_10_ emulsification is greatly reduced due to the screening of electrostatic interactions. To increase the solubilization efficiency, octane-1,2-diol was replaced by longer chain 1,2-diols; however, this triggered the formation of highly viscous liquid crystalline phases due to the ionic nature of the di-rhamnolipid. Instead, using partially protonated Rha_2_C_10_C_10_ at pH ∼ p*K*_a_ and a fixed salinity ϵ = 0.030, the use of decane-1,2-diol and dodecane-1,2-diol led to a progressively increasing solubilization efficiency, which can be explained by the resulting increase in the bare bending rigidity of the mixed amphiphilic film.

Investigations of the nanostructure with SANS revealed the formation of well-structured microemulsions with values of the amphiphilicity factor *f*_a_ around −0.7 (except for the salt-free system (ϵ = 0.000) at pH ≫ p*K*_a_ containing very high fractions of octane-1,2-diol). Well-structured bicontinuous microemulsions, stabilized by an interfacial film, feature ultralow interfacial tensions and excellent wetting properties that are beneficial for, e.g., washing applications. Other applications include their use as a carrier for therapeutic molecules like antimicrobial peptides^78^ or proteins.^79^ In contrast, systems stabilized by biobased cardanol/alcohol mixtures were found to show predominantly positive *f*_a_ values, indicating the formation of loosely ordered and highly dynamic structures, similar to so-called surfactant-free microemulsions,^19^ which can be used, e.g., as dispersants for crude oil.^80^

The analysis of the structure peak in our rhamnolipid-stabilized microemulsions showed that, in accordance with the phase diagram studies, the largest domain size, *d*_TS_/2 = 156 Å, is found in the salt-free system (ϵ = 0.000) and at pH ≫ p*K*_a_, where the anionic nature of Rha_2_C_10_C_10_ leads to a minimum in the required amount of the surfactant. Smaller domain sizes around *d*_TS_/2 = 40 – 45 Å were found upon addition of KCl, consistent with the reduced efficiency of the Rha_2_C_10_C_10_/octane-1,2-diol mixture. The use of partially protonated Rha_2_C_10_C_10_ at pH ∼ p*K*_a_ in combination with the longer-chain dodecane-1,2-diol enabled the stabilization of water and IPM domains with a larger length scale of *d*_TS_/2 = 99 Å.

Striking and unexpected features of these di-rhamnolipid microemulsions were uncovered by the analysis of the high-*q* region of the scattering curves. Using the modified Porod model to describe these data, we found surprisingly low values of *t* in the Gaussian term (eq 9) that models the blurry nature of the amphiphilic film. Given the size of the hydrated double sugar headgroup of Rha_2_C_10_C_10_, larger values of *t* would have been expected. We suspect that the contribution of the bulky sugar head groups to the local SLD profile gives rise to an additional scattering contribution^66–68^ that partially compensates the Gaussian term in eq 9. Furthermore, we believe that this additional scattering contribution of the sugar head groups also explains the geometrical factor of *a* = 10.6, which is significantly larger than the value predicted by geometrical models of the bicontinuous structure^49,73^ and found experimentally for microemulsions stabilized by nonionic alkyl polyglycolether surfactants.^50^ Finally, for the microemulsions at pH ≫ p*K*_a_, where the di-rhamnolipid is fully deprotonated, giving rise to ionic interactions, we observed an additional scattering contribution at even higher *q*-values. While the exact nature of this scattering contribution remains unknown as of today, we have good reasons to believe that it is most likely linked to the protonated rhamnose moieties whose packing conformation is known to depend on pH and ionic strength.^76^ A second possible origin of this scattering feature could lie in the interfacial segregation of Rha_2_C_10_C_10_ due to electrostatic repulsion between neighboring rhamnolipid molecules. We plan to investigate this phenomenon in an upcoming work, combining contrast variation SANS with molecular dynamics simulations.

In conclusion, we showed in this contribution that symmetric microemulsions stabilized by the biosurfactant di-rhamnolipid and alkane-1,2-diols are well-structured, especially in the presence of small amounts of salt, allowing for the screening of the electrostatic headgroup repulsions. Our results thus provide fundamental insights into the properties of rhamnolipid-containing, pseudoquaternary microemulsion systems that are especially interesting for applications where mildness and biocompatibility of the compounds are required.

## Supplementary Material



## Data Availability

The data can be downloaded free of charge from the data repository of the University of Stuttgart (DaRUS) via the following DOI: https://doi.org/10.18419/DARUS-6332. SANS measurement data, obtained at the Institut Laue Langevin, are available at the ILL data repository 10.5291/ILL-DATA.9-10-1900

## Notes

Sample compositions, summary of the coordinates of all *X̃*-points, brief analysis of the effects of multiple scattering on the SANS curves, schematic depiction of the Kahlweit fish cut through the phase tetrahedron, demonstration of the influence of background subtraction and different blurriness parameters *t* on the high-*q* analysis, and additional SANS measurements at high co-surfactant fractions (PDF)
