# Supplementary material for: General Patterns of Biocompatible Microemulsions Formed with Di-rhamnolipid and Alkanediols
Source: Langmuir. 2026 Jul 25;42(31):22994–3006. doi: 10.1021/acs.langmuir.6c03072 (PMC13472193; doi:10.1021/acs.langmuir.6c03072)
Supplement: Supplementary file 1 [file la6c03072_si_001.pdf]

Supporting Information:

General patterns of biocompatible microemulsions formed with di-rhamnolipid and alkanediols

Florian Trummer,[†] Rika Raff,[†] Sylvain Prévost,[‡] and Thomas Sottmann[*,†]

†*Institute of Physical Chemistry, University of Stuttgart, Pfaffenwaldring 55, 70569 Stuttgart, Germany*

‡*Institut Laue-Langevin – The European Neutron Source, 71 Avenue des Martyrs, F-38042 Grenoble, France*

E-mail: thomas.sottmann@ipc.uni-stuttgart.de

Phone: +49 (0)711 685-64494

Table of Contents

S1 $\tilde{X}$-point coordinates obtained from phase diagram measurements **S-3**

S2 3-phase equilibrium in salt-free microemulsions ($\epsilon = 0.000$) **S-4**

S3 Sample composition SANS **S-5**

S4 Multiple Scattering Effects and data treatment with the MuScatt software **S-5**

S5 Schematic depiction of the Kahlweit fish cut **S-9**

S6 Data analysis with modified Porod law **S-10**

S7 Influence of high co-surfactant content in the amphiphile mixture on the microstructure **S-11**

S1 $\tilde{X}$-point coordinates obtained from phase diagram measurements

This section summarises the coordinates of the $\tilde{X}$-points ('efficiency points') as obtained from the phase diagrams presented in the main manuscript. In pseudo-quaternary, symmetric ($\phi = 0.5$) microemulsions, consisting of H_2O/Brine(A) – Oil(B) – Surfactant(C) – Co-surfactant(D), the coordinates of the $\tilde{X}$-point are the minimum surfactant concentration ($\tilde{\gamma}_C$) and co-surfactant concentration ($\tilde{\gamma}_D$) required for the formation of a one-phase microemulsion, and hence full solubilisation of the aqueous and oil phase.

Displayed in Tab. S1 are the $\tilde{X}$-points found for microemulsions formulated in the pseudo-quaternary system H_2O/KCl-Brine – Isopropylmyristate – $Rha_2C_{10}C_{10}$ (pH = $(8.0 \pm 0.2) \gg$ pKa) – Octane-1,2-diol at $T = 35\,°C$ with varying salt concentration ϵ of the aqueous phase.

Table S1: Coordinates ($\tilde{\gamma}_C$, $\tilde{\gamma}_D$) of the $\tilde{X}$-points of symmetric ($\phi = 0.5$) microemulsions at varying salt concentration ϵ in the pseudo-quaternary system H_2O/KCl-Brine – Isopropylmyristate – $Rha_2C_{10}C_{10}$ (pH = 8.0) – Octane-1,2-diol at $T = 35\,°C$. The values were obtained from the phase diagrams displayed in Fig. 2 of the main manuscript.

ϵ	$\tilde{\gamma}_C$	$\tilde{\gamma}_D$
0.000	0.025	0.110
0.010	0.095	0.160
0.030	0.120	0.170
0.050	0.145	0.175
0.100	0.160	0.155

Table S2 comprises the $\tilde{X}$-points found for microemulsions formulated in the pseudo-quaternary system KCl-Brine ($\epsilon = 0.030$) – Isopropylmyristate – $Rha_2C_{10}C_{10}$ – Octane-1,2-diol at $T = 35\,°C$ formulated with rhamnolipid at pH = $(8.0 \pm 0.2) \gg$ pKa and pH = $(6.0 \pm 0.5) \sim$ pKa respectively.

Table S2: Coordinates ($\tilde{\gamma}_C$, $\tilde{\gamma}_D$) of the $\tilde{X}$-points of symmetric ($\phi = 0.5$) microemulsions at varying pH in the pseudo-quaternary system KCl-Brine ($\epsilon = 0.030$) – Isopropylmyristate – $Rha_2C_{10}C_{10}$ – Octane-1,2-diol at $T = 35\,°C$. The values were obtained from the phase diagrams displayed in Fig. 3 (left) of the main manuscript.

pH	$\tilde{\gamma}_C$	$\tilde{\gamma}_D$
8.0	0.120	0.170
6.0	0.155	0.130

Finally, Tab. S3 summarises the $\tilde{X}$-points found for microemulsions formulated in the pseudo-quaternary system KCl-Brine ($\epsilon = 0.030$) – Isopropylmyristate – $Rha_2C_{10}C_{10}$ (pH = $(6.0 \pm 0.5) \sim$ pKa) – C_k-1,2-diol at $T = 35\,°C$ for different co-surfactant alkyl chain lengths

k.

Table S3: Coordinates ($\tilde{\gamma}_C$, $\tilde{\gamma}_D$) of the $\tilde{X}$-points of symmetric ($\phi = 0.5$) microemulsions in the quaternary system KCl-Brine ($\epsilon = 0.030$) – Isopropylmyristate – $Rha_2C_{10}C_{10}$ (pH = 6.0) – C_k-1,2-diol with varying co-surfactant alkyl chain length k at $T = 35\,°C$. The values were obtained from the phase diagrams displayed in Fig. 3 (right) of the main manuscript.

k	$\tilde{\gamma}_C$	$\tilde{\gamma}_D$
8	0.155	0.130
10	0.135	0.080
12	0.105	0.065

S2 3-phase equilibrium in salt-free microemulsions ($\epsilon = 0.000$)

In Fig. S1, we show the three-phase coexistence between an L_α, a microemulsion and an oil excess phase in a phase-separated sample formulated at a surfactant concentration $\gamma_C = 0.034$, a co-surfactant concentration $\gamma_D = 0.116$ without added salt ($\epsilon = 0.000$) at $T = 20\,°C$. The photo without optical polarisers (left) highlights the existence of a light-scattering microemulsion middle phase and an oil excess phase that does not scatter the green laser. The existence of the bottom L_α phase is confirmed via inspection of the sample between crossed optical polarisers (right).

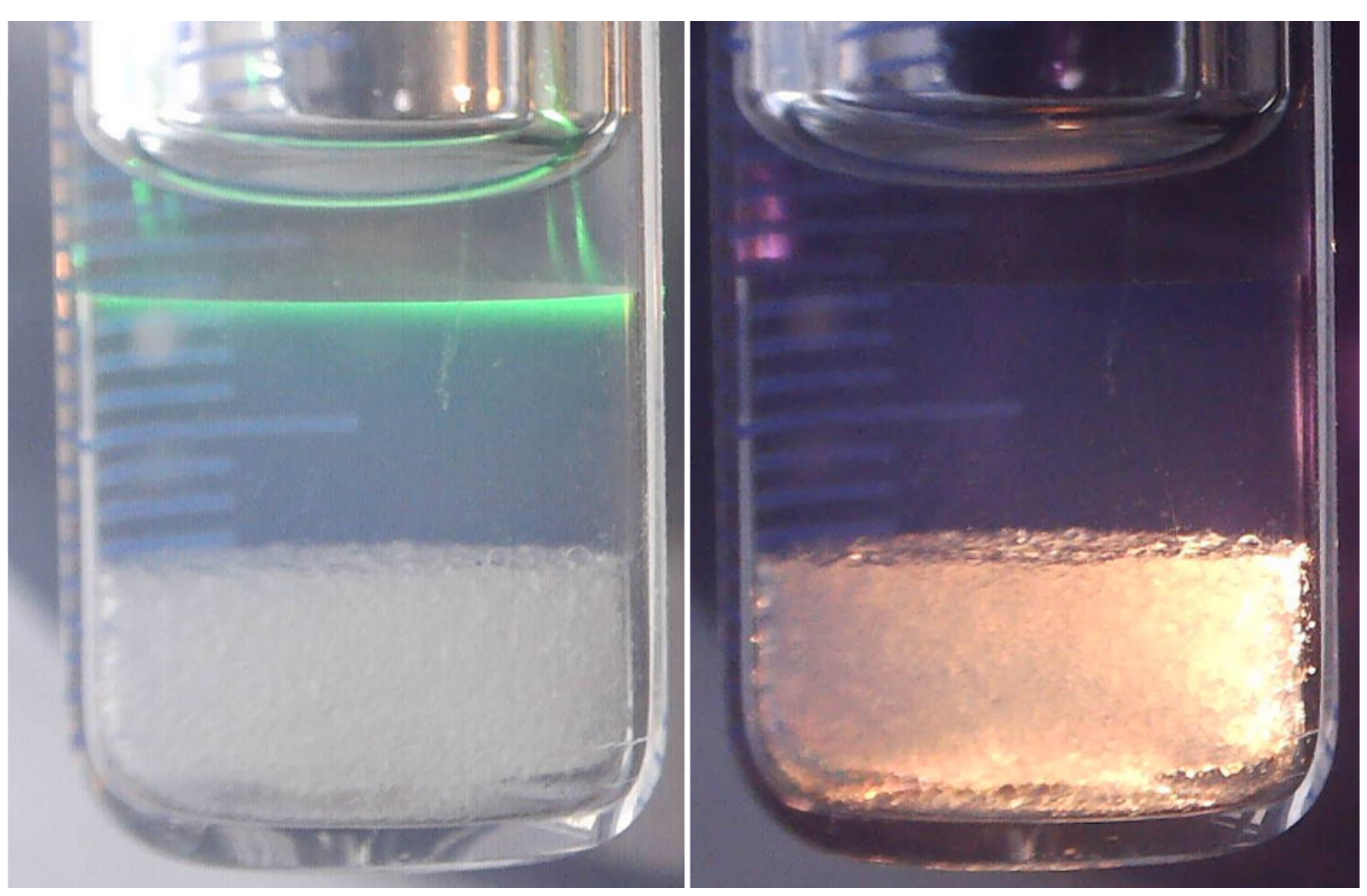

Figure S1: Phase-separated sample in the quaternary system H_2O ($\epsilon = 0.000$) – Isopropylmyristate – $Rha_2C_{10}C_{10}$ (pH = 8.0) – Octane-1,2-diol with $\gamma_C = 0.034$ and $\gamma_D = 0.116$ at $T = 20\,°C$. Shown is (left) the sample without polarisers with a laser illuminating the sample from the side and (right) the same sample between crossed linear polarisers.

S3 Sample composition SANS

This section summarises the composition of microemulsion samples for SANS measurements, where D_2O was used instead of H_2O in order to reach bulk contrast conditions between the deuterated aqueous phase and the protonated oil phase.

Table S4: Composition of SANS microemulsion samples in the pseudo-quaternary system D_2O/KCl brine (A) – Isopropylmyristate (B) – $Rha_2C_{10}C_{10}$ (pD = 8.0) (C) – Octane-1,2-diol (D) with different salt concentrations ϵ of the aqueous phase.

ϵ	m_A / mg	m_B / mg	m_C / mg	m_D / mg
0.000	569.7	436.2	34.2	159.8
0.010	456.2	347.5	166.5	229.7
0.030	454.9	342.7	172.4	229.9
0.050	450.4	335.6	184.0	230.0
0.100	440.5	319.4	208.5	231.6

Table S5: Composition of SANS microemulsion samples in the pseudo-quaternary system D_2O/KCl brine (ϵ = 0.030) (A) – Isopropylmyristate (B) – $Rha_2C_{10}C_{10}$ (C) – Octane-1,2-diol (D) with pD = $(6.0 \pm 0.5) \approx$ pKa and pD = $(8.0 \pm 0.2) \gg$ pKa respectively. The sample with pD = (8.0 ± 0.2) is the same as in Tab. S4 and given again for the sake of clarity.

pD	m_A / mg	m_B / mg	m_C / mg	m_D / mg
8.0	454.9	342.7	172.4	229.9
6.0	472.7	356.1	207.5	163.7

Table S6: Composition of SANS microemulsion samples in the pseudo-quaternary system D_2O/KCl brine (ϵ = 0.030) (A) – Isopropylmyristate (B) – $Rha_2C_{10}C_{10}$ (pD = 6.0) (C) – C_k-1,2-diol (D) with different co-surfactant alkyl chain length k. The sample with $k = 8$ is the same as in Tab. S5 and given again for the sake of clarity.

k	m_A / mg	m_B / mg	m_C / mg	m_D / mg
8	472.7	356.1	207.5	163.7
10	514.7	387.7	189.6	108.0
12	543.9	409.7	154.7	91.6

S4 Multiple Scattering Effects and data treatment with the MuScatt software

We investigated the influence of multiple scattering on the SANS spectra by measuring the salt-free sample (ϵ = 0.000) at three different wavelengths, 4.6, 6 and 8 Å. The resulting scattering curves are displayed in Fig. S2.

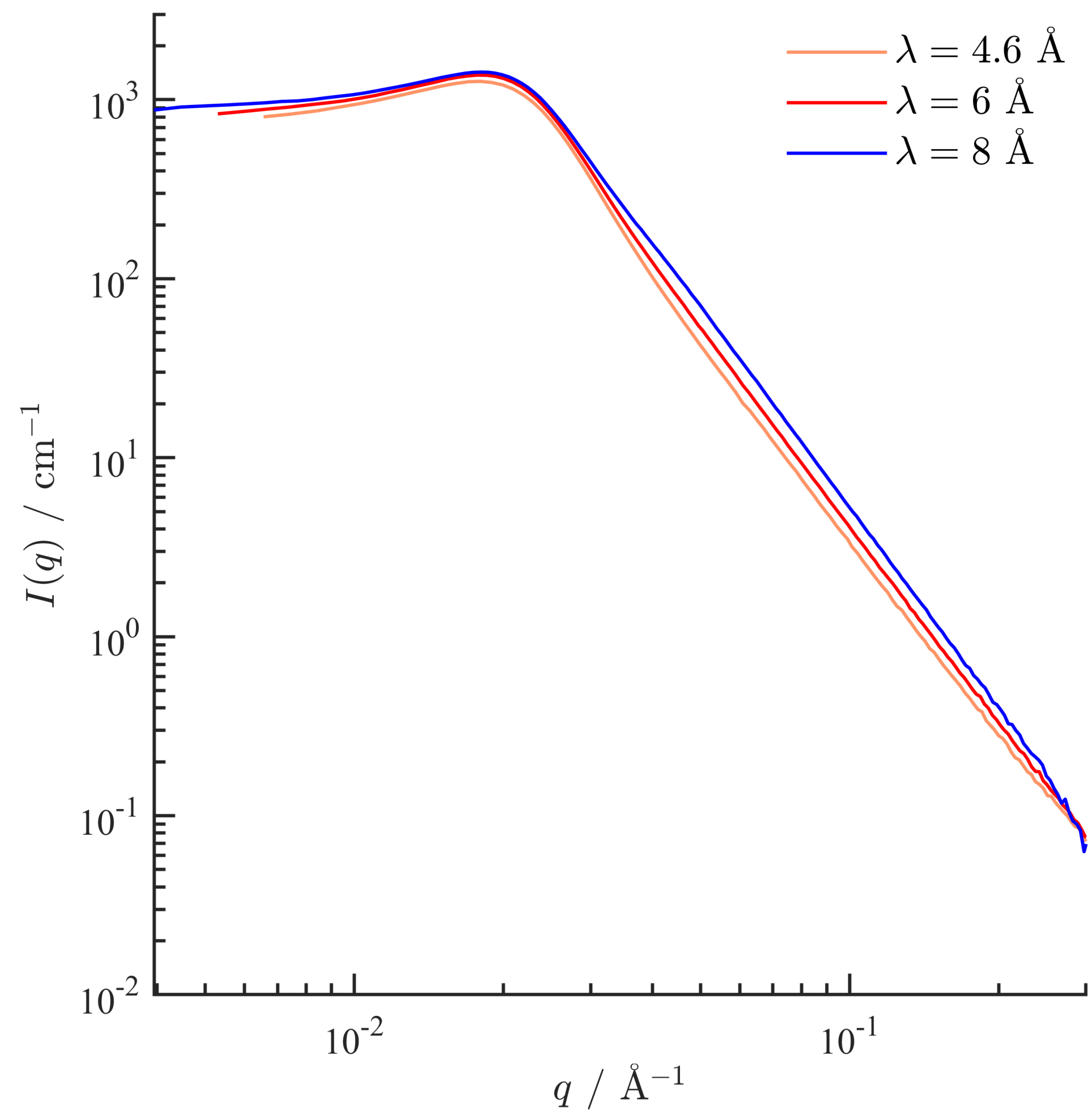

Figure S2: SANS data of the salt-free sample ($\epsilon = 0.000$) measured at different neutron wavelengths. The data are displayed as lines for better visualisation

As expected, multiple scattering occurs at larger wavelengths and noticeably affects the width of the correlation peak, the absolute scattering intensity and the slope of the scattering curve in the region between the peak position q_{max} and $2q_{\mathrm{max}}$.

As described in the main manuscript, the scattering data of the salt-free ($\epsilon = 0.000$) microemulsion sample, collected at $\lambda = 4.6$ Å, was processed with the MuScatt algorithm that allows to at least partially remove multiple scattering effects. The scattering curves and the associated Porod plots before and after this data treatment are shown in Fig. S3 left and right respectively.

In this representation, the effect of the MuScatt treatment on the peak width and the adjacent slope of the scattering curve can be clearly seen. However, the broad additional scattering feature at high q is not removed by the data processing and can not be explained

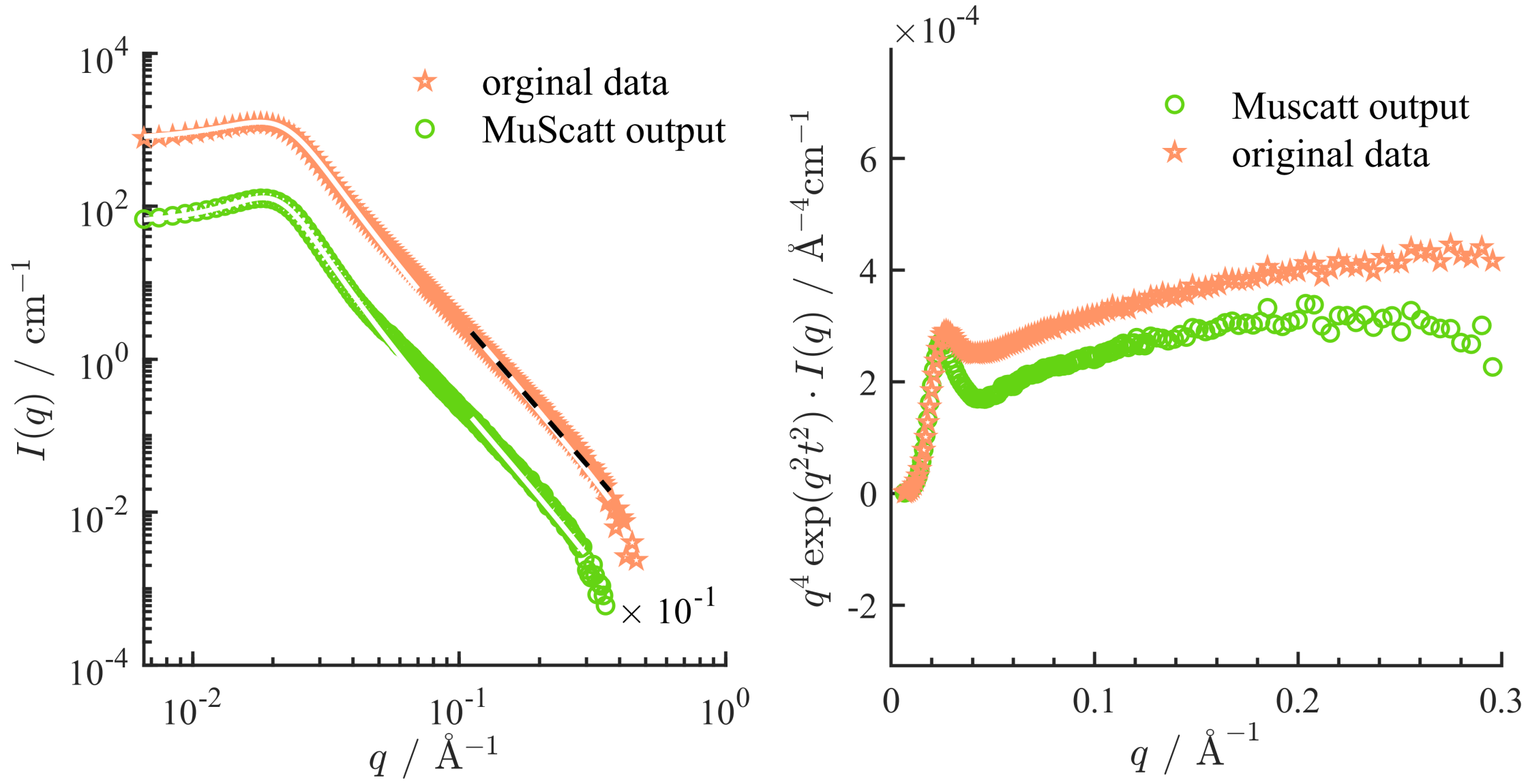

Figure S3: Comparison of the SANS curve of the salt-free sample ($\epsilon = 0.000$) before and after the treatment with the MuScatt software. On the (left), the original and MuScatt-treated data are displayed together with the respective fits. The white lines give the fits with the combination of the Teubner-Strey model and the modified Porod model ($t = 1.5$ Å) as defined in the main mansucript. The dashed black fit line shows the high-q fit with the classic Porod model for infinitely sharp interfaces ($t = 0$ Å) for comparison.

by multiple scattering.

Table S7: Fit parameters from the analysis of the SANS curves shown in Fig. 7 of the main mnuscript. We compare the results for the salt-free sample ($\epsilon = 0.000$) before and after the treatment with the MuScatt software. For the untreated data, the comparison for the fit with a sharp ($t = 0$ Å) and a blurry interface $t = 1.5$ Å is made. Shown are the domain size, $d_{\mathrm{TS}}/2$ and correlation length, ξ_{TS} from the TS-model along with the invariant Q calculated from the second moment of the scattering intensity and the specific internal interface from the Porod analysis, (S/V), including the width of the blurry interface t. The amphiphilicity factor is denoted by f_{a}.

ϵ	$d_{\mathrm{TS}}/2$ / Å	ξ_{TS} / Å	Q / cm^{-1}Å^{-3}	(S/V) / Å^{-1}	t / Å	f_{a}
Original data ($t = 0$ Å)	156	108	0.0171	0.0158	0.0	-0.65
Original data ($t = 1.5$ Å)	156	108	0.0165	0.0169	1.5	-0.65
MuScatt output	158	131	0.0138	0.0154	1.5	-0.74

As can be seen, the removal of the multiple scattering effects has a strong influence on the correlation length ξ_{TS} and the amphiphilicity factor f_{a} that is directly linked to it. Furthermore, the invariant Q is slightly lowered due to a lower total scattering intensity in the post-treatment data. Finally, the final slope of the MuScatt-treated curve is best described by a modified Porod law with a blurry interface of thickness $t = 1.5$ Å. The value for t after the MuScatt treatment is in accordance with the values found for all other scattering curves

shown in the main manuscript.

S5 Schematic depiction of the Kahlweit fish cut

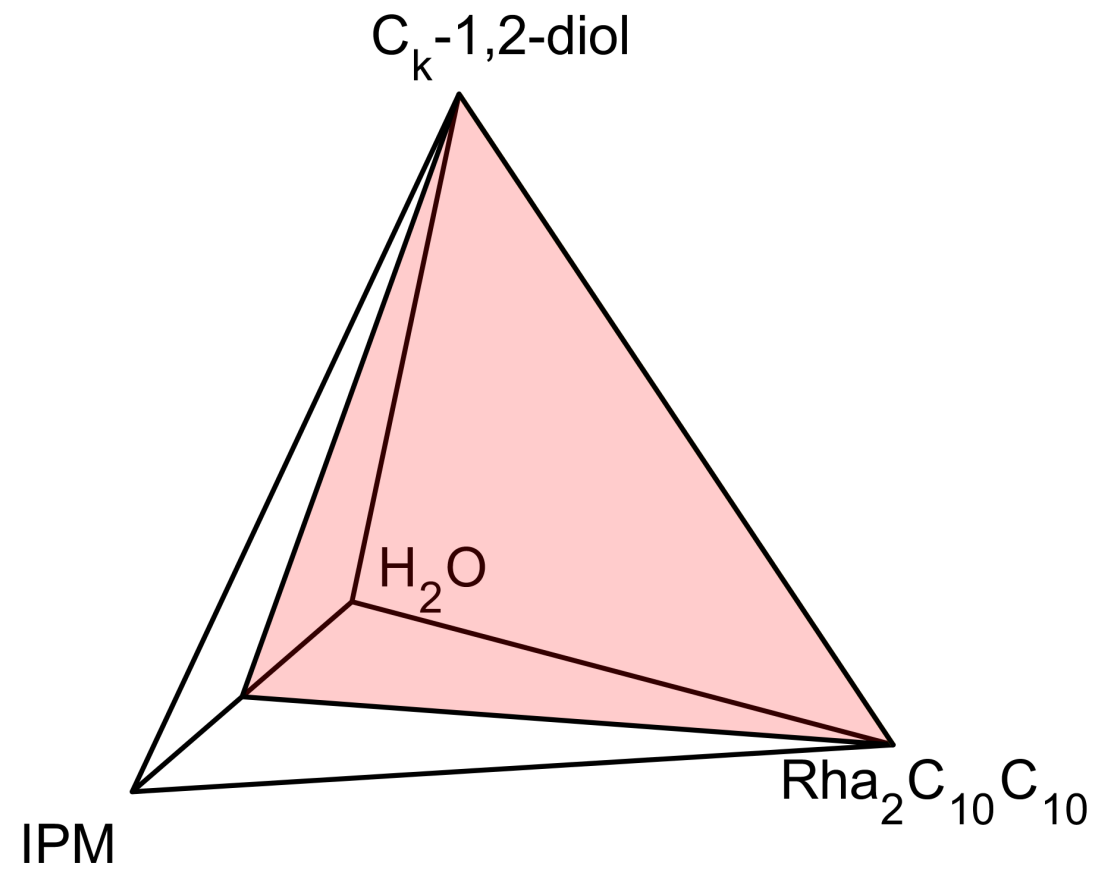

Figure S4: Schematic depiction of the Kahlweit fish cut ($\phi = 0.5$) through the phase tetrahedron in the quaternary system H_2O – IPM – $Rha_2C_{10}C_{10}$ – $C_k - 1, 2 -$ diol.

S6 Data analysis with modified Porod law

In order to validate our high-q analysis with the modified Porod law (Eq. 9 in the main manuscript), we show in Fig. S5 (left) the influence of different values for the incoherent background subtraction for one exemplary SANS curve of the microemulsion sample formed by D_2O(KCl, $\epsilon = 0.030$) – IPM – $Rha_2C_{10}C_{10}$ (pH $\sim$ pKa) – C_{12}-1,2-diol. As can be seen, a slightly too small value for the background (Bkg $= 0.63\,cm^{-1}$) leads to a residual plateau at high q, while using a slightly too large value (Bkg $= 0.65\,cm^{-1}$) results in a loss of data points. In Fig. S5 (right), we show for the latter background value, that various values of the blurriness t cannot accurately describe the high q part of the data. The optimum values used in the manuscript (Bkg $= 0.64\,cm^{-1}$ and $t = 2$ Å) give the best fit to the data.

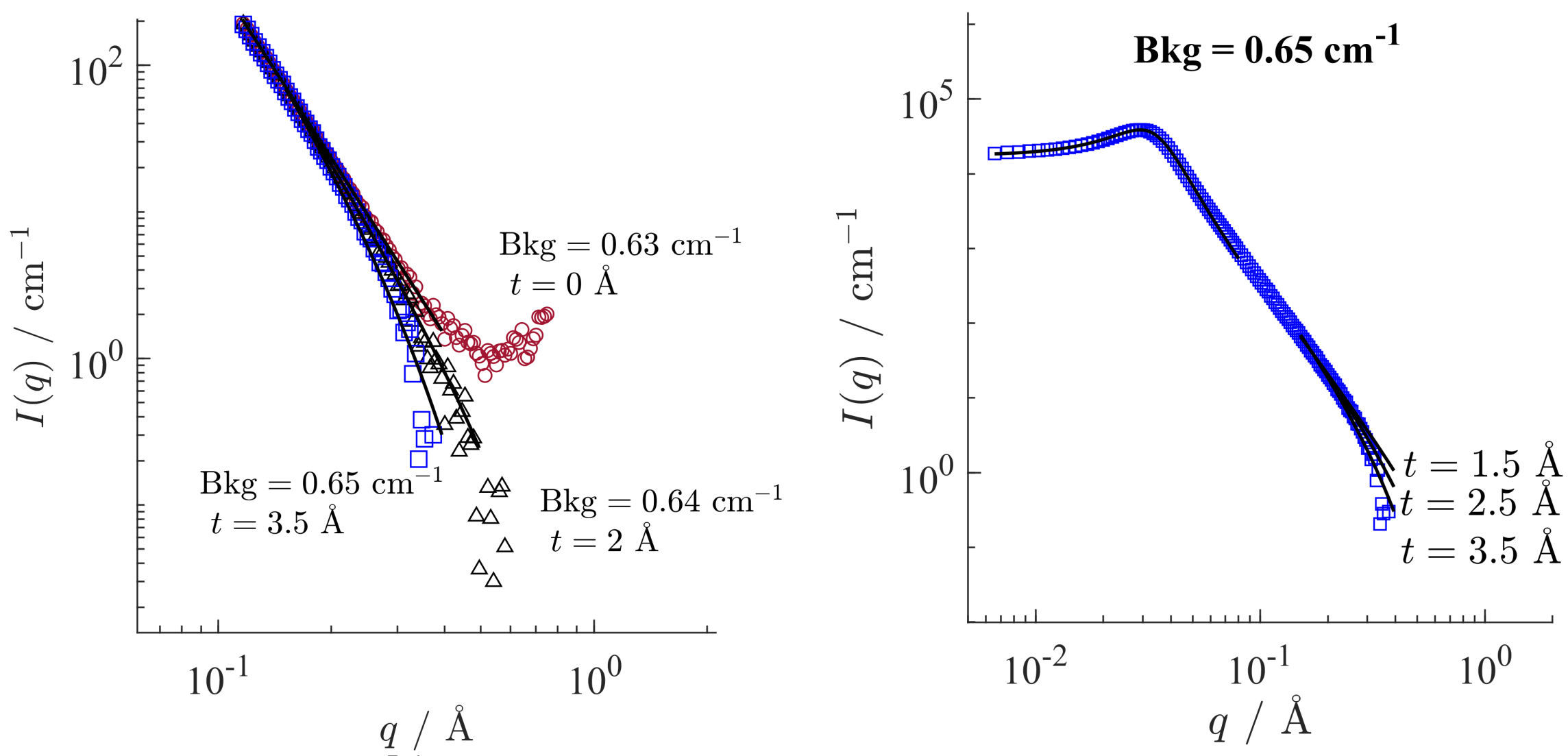

Figure S5: Bulk contrast SANS curve of the microemulsion sample ($\phi = 0.50$) formed by D_2O(KCl, $\epsilon = 0.030$) – IPM – $Rha_2C_{10}C_{10}$ (pH $\sim$ pKa) – C_{12}-1,2-diol recorded close to the $\tilde{X}$-point at 35 °C. On the left, different values for the incoherent background (Bkg) were subtracted from the original data and a modified Porod law was fitted with the respective optimum blurriness t of the interfacial film, indicated in the respective label. On the (right), it is shown that using the slightly exaggerated background value (0.65 cm^{-1}), cannot be compensated by high values of the blurriness t.

S7 Influence of high co-surfactant content in the amphiphile mixture on the microstructure

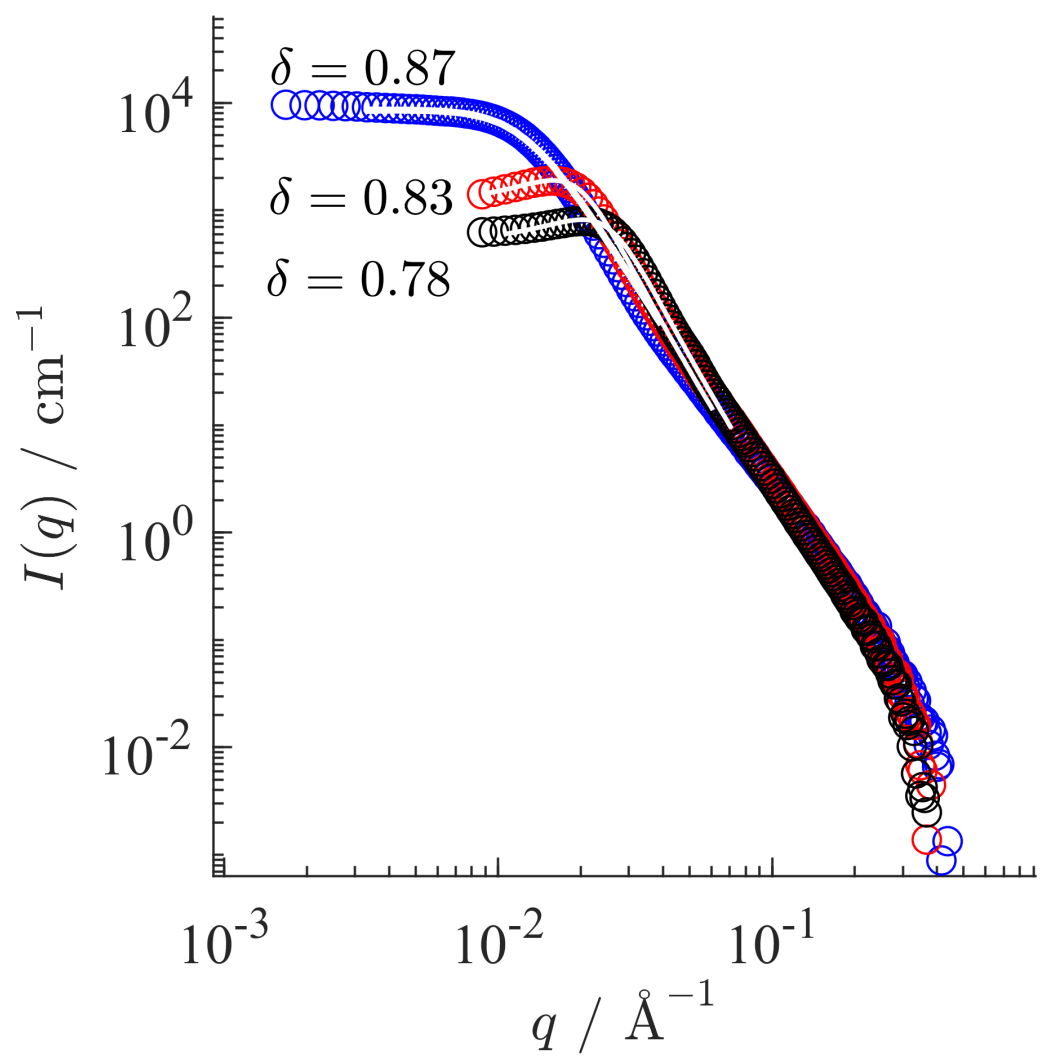

Figure S6: Bulk contrast SANS curves and corresponding Teubner-Strey fits of the salt-free ($\epsilon = 0.000$) microemulsion sample ($\phi = 0.50$) formed by D_2O – IPM – $Rha_2C_{10}C_{10}$ (pH $\gg$ pKa) – octane-1,2-diol at a fixed total amphiphile concentration $\gamma = 0.15$ and various fractions δ of octane-1,2-diol in the amphiphile mixture, recorded at 30 °C.

Displayed in Fig. S6 are the bulk-contrast SANS curves of salt-free ($\epsilon = 000$), symmetric ($\phi = 0.50$) microemulsions formed by D_2O – IPM – $Rha_2C_{10}C_{10}$ (pH $\gg$ pKa) – octane-1,2-diol at 30 °C. The total amphiphile concentration $\gamma = \gamma_C + \gamma_D = 0.15$ is kept constant, while the relative fraction of octane-1,2 in the amphiphile mixture $\delta = \gamma_D/\gamma$ is varied between 0.78 and 0.87. As can be seen, the correlation peak moves to lower q with increasing δ, indicating a growth of the water/oil domains. In addition, a flattening of the peak is observed at the highest co-surfactant fraction $\delta = 0.87$, confirming a less ordered structure in the microemulsions.

The structural parameters obtained from the analysis of the correlation peak with the Teubner-Strey model are summarised in Tab. S7 together with those of the sample presented in the main manuscript, which was prepared at a slightly higher total amphiphile concentration $\gamma = 0.17$. At a constant $\delta = 0.83$, a decrease of the total amphiphile concentration γ from 0.17 to 0.15 leads to an increase of the domain size $d_{TS}/2$ from 176 to 156 Å, while the amphiphilicity factor f_a stays constant at -0.65.

Upon increasing the amount of octane-1,2-diol in the amphiphile mixture at a constant $\gamma = 0.15$ and thus approaching the upper phase boundary of the one-phase region (see Fig. 1 (left) in the main manuscript), a growth in the domain size $d_{TS}/2$ from 137 Å ($\delta = 0.78$),

to 176 Å ($\delta = 0.83$), and finally 342 Å ($\delta = 0.87$) is observed. In addition, the amphiphilicity factor f_a decreases from values around -0.67 to -0.09. While a value of $f_a < -0.6$ is typical for well-structured microemulsion, the increase of f_a to values close to 0 indicates a loss of order in the microemulsion structure, as the upper phase boundary is approached. The near-zero value of f_a for $\delta = 0.87$ could indicate the vicinity to a Lifshitz line.

Table S8: Structural parameters obtained from the analysis of the correlation peaks of the SANS curves shown in Fig. S6 with the Teubner-Strey model. For reference, the parameters from the sample presented in the main manuscript, prepared at a slightly higher total amphiphile concentration γ are shown (top grey row). Displayed are the total amphiphile concentration γ, the fraction of co-surfactant in the amphiphile mixture δ, the domain size $d_{TS}/2$, the correlation length ξ_{TS} and the amphiphilicity factor f_a.

γ	δ	$d_{TS}/2$ (Å)	ξ_{TS} (Å)	f_a	T / °C
0.17	0.83	156	108	−0.65	35
0.15	0.78	137	99	−0.67	30
0.15	0.83	176	121	−0.65	30
0.15	0.87	342	119	−0.09	30
